# Online breath analysis with SESI/HRMS for metabolic signatures in children with allergic asthma

**DOI:** 10.3389/fmolb.2023.1154536

**Published:** 2023-03-31

**Authors:** Ronja Weber, Bettina Streckenbach, Lara Welti, Demet Inci, Malcolm Kohler, Nathan Perkins, Renato Zenobi, Srdjan Micic, Alexander Moeller

**Affiliations:** ^1^ Department of Respiratory Medicine, University Children’s Hospital Zurich, Zurich, Switzerland; ^2^ Department of Chemistry and Applied Biosciences, ETH Zurich, Zurich, Switzerland; ^3^ Department of Pulmonology, University Hospital Zurich, Zurich, Switzerland; ^4^ Division of Clinical Chemistry and Biochemistry, University Children’s Hospital Zurich, Zurich, Switzerland

**Keywords:** volatile organic compounds (VOCs), metabolites, allergic asthma, children, breath analysis, SESI/HRMS

## Abstract

**Introduction:** There is a need to improve the diagnosis and management of pediatric asthma. Breath analysis aims to address this by non-invasively assessing altered metabolism and disease-associated processes. Our goal was to identify exhaled metabolic signatures that distinguish children with allergic asthma from healthy controls using secondary electrospray ionization high-resolution mass spectrometry (SESI/HRMS) in a cross-sectional observational study.

**Methods:** Breath analysis was performed with SESI/HRMS. Significant differentially expressed mass-to-charge features in breath were extracted using the empirical Bayes moderated t-statistics test. Corresponding molecules were putatively annotated by tandem mass spectrometry database matching and pathway analysis.

**Results:** 48 allergic asthmatics and 56 healthy controls were included in the study. Among 375 significant mass-to-charge features, 134 were putatively identified. Many of these could be grouped to metabolites of common pathways or chemical families. We found several pathways that are well-represented by the significant metabolites, for example, lysine degradation elevated and two arginine pathways downregulated in the asthmatic group. Assessing the ability of breath profiles to classify samples as asthmatic or healthy with supervised machine learning in a 10 times repeated 10-fold cross-validation revealed an area under the receiver operating characteristic curve of 0.83.

**Discussion:** For the first time, a large number of breath-derived metabolites that discriminate children with allergic asthma from healthy controls were identified by online breath analysis. Many are linked to well-described metabolic pathways and chemical families involved in pathophysiological processes of asthma. Furthermore, a subset of these volatile organic compounds showed high potential for clinical diagnostic applications.

## 1 Introduction

Asthma is the most frequent chronic condition in children in the developed world. The disease is very heterogeneous in its presentation and clinical course. Due to the lack of a well-recognized and easy to apply diagnostic gold-standard (Gaillard et al., 2021), misdiagnosis is relatively common. Reported numbers range from 10% to 62% for underdiagnosis (Kaur et al., 1998; Siersted et al., 1998; van Gent et al., 2007) from 48% to 53% for overdiagnosis (Looijmans-van den Akker et al., 2016; Yang et al., 2017). This has negative impacts on asthma related morbidity, quality-of-life, medication side-effects, prognosis, and health costs. Therefore, the investigation of pediatric asthma and its associated molecular processes including airway inflammation is of high importance for the development of novel, much-needed diagnostic and monitoring applications.

Breath is known to contain several hundreds of metabolites that reflect metabolism as well as disease-specific mechanisms such as airway inflammation (Ferraro et al., 2018). Therefore, there is great interest in discovering endogenous exhaled organic compounds that are linked to diseases and their pathophysiological processes (Neerincx et al., 2017). One of the few clinical tests taking advantage of this is the quantification of exhaled fractional nitric oxide (FeNO), which can be measured in all age groups. FeNO is a biomarker for eosinophilic airway inflammation that is related to allergic asthma (Ferraro et al., 2018). This exemplifies the potential of applying breath analysis to further study allergic asthma and improve the diagnostic power of exhaled biomarkers.

Several breath analysis studies attempted to distinguish children with asthma from healthy controls by different techniques. Dallinga and colleagues compared exhaled breath of children with asthma and a healthy group by gas chromatography mass spectrometry and identified a small set of discriminatory volatile organic compounds (VOCs) that is potentially related to lipid peroxidation, including various hydrocarbons, xylene, benzoic acid, and butanoic acid (Dallinga et al., 2010). A pilot study from van Mastrigt et al. identified VOCs discriminating children with asthma, cystic fibrosis and healthy controls by using a broadband quantum cascade laser spectroscopy technique (van Mastrigt et al., 2016). The distinguishing compound classes included different carboxylic acids, esters, and ethers. Altogether, there is only little overlap between the detected metabolites of different studies and sometimes even conflicting results are reported. Therefore, standardization as well as external validation are challenges that need further research as summarized in recent reviews (Neerincx et al., 2017; Ferraro et al., 2018; Papamichael et al., 2021).

Secondary electrospray ionization high-resolution mass spectrometry (SESI/HRMS) is a technology applied for online breath analysis that links real-time measurements without sample preparation to high mass resolution (Gaugg et al., 2019). The latter strongly improves the confidence in compound identification of the detected mass-to-charge features (*m/z* features). Previous studies confirmed the potential of this technology to identify relevant exhaled organic compounds, including biological metabolites, and reveal altered molecular pathways for different respiratory diseases (Schwarz et al., 2016; Gaugg et al., 2019; Weber et al., 2020). A strength of SESI/HRMS lies in the detection of polar molecules with high molecular masses and low volatility (Gaugg et al., 2016; Bruderer et al., 2019; Chen et al., 2021). Furthermore, its applicability in children was confirmed in our previous study on cystic fibrosis (Weber et al., 2020).

The aim of this study was to identify metabolic signatures in exhaled breath consisting of discriminating organic compounds specific to allergic asthma in children by SESI/HRMS and to assess their biological context.

## 2 Materials and methods

### 2.1 Study design, participants, and clinical data

This observational cross-sectional study included children with allergic asthma and healthy controls, aged 5–18 years. Asthmatic patients from the outpatient clinic of the University Children’s Hospital Zürich, Switzerland, were recruited for this study. Asthma diagnosis was based on the recent ERS evidence-based practice guidelines (Gaillard et al., 2021) and only children with confirmed asthma were included. Allergic sensitization was defined by either a positive skin prick test or an allergen-specific IgE of >0.35 kU·L–1 by radioallergosorbent test or by ELISA for at least one common aeroallergen. Further, eligible patients were clinically stable enough to temporarily stop the inhalation of long-acting asthma medication at least 1 week before the measurements. Exclusion criteria were the inability to stop medication, and the presence of an acute respiratory infection during the last 2 weeks before the measurement. Clinical data was collected on the same day as breath analysis and is summarized together with anthropometric data in Table 1. Healthy controls without any chronic respiratory symptoms or known lung diseases were recruited from the public. The presence of an acute (respiratory) infection was an exclusion criterion for both groups. The measurement and recruitment period were in parallel and lasted for 15 months. Efforts were put into recruiting participants of both cohorts at a randomized schedule across daytime and throughout the study period. The sample size was based on our previous study with a similar design (Weber et al., 2020). All participants, where appropriate and parents gave their written informed consent in advance. The study was approved by the local ethics committee (KEK-ZH ID 2018–00441) and was conducted in accordance with the Declaration of Helsinki.

**TABLE 1:** Participant characteristics.

	Allergic asthma (n = 48)	Healthy controls (n = 56)	*p*-value
Age [y]	12.1 ± 3.1	10.8 ± 4.0	0.07
Male sex [n]	33 (68.8%)	24 (42.9%)	0.01
BMI [kg/m^2^]	19.3 ± 4.2	18.3 ± 3.3	0.2
FEV1 [z-score]	−0.6 ± 1.1	−0.1 ± 1.0 ^†^	0.01
FVC [z-score]	0.1 ± 1.0	0.1 ± 0.9 ^†^	0.86
FeNO [ppb]	28.6 IQR 34.4	6.2 IQR 9.6	<0.001
Allergic sensitization [n]	48 (100%)	12 (21.4%)	<0.001

Data are presented as mean ± standard deviation (SD), n (%), or median and interquartile range (IQR). BMI, body mass index, pre-bronchodilator FEV1 = forced expiratory volume in 1 s, pre-bronchodilator FVC, forced vital capacity, FeNO, fractional exhaled nitric oxide. *p*-values were determined by the two sample *t*-test, Fisher’s exact test for sex and allergic sensitization distribution, and the Mann-Whitney U test for FeNO, values (no normal distribution). †: 16 spirometries were excluded because of poor quality.

### 2.2 Breath analysis

Online breath analysis was performed using a SESI source (SuperSESI, FIT FossilionTech, Madrid, Spain) connected to a high-resolution time-of-flight mass spectrometer (TripleTOF 5600+, AB Sciex, Concord, ON, Canada). Methodological details and instrumental settings were previously described by our group (Weber et al., 2020). Minor adaptations are specified below. Children were exhaling directly into the instrument in a sitting position. The breathing maneuver consisted of at least three long exhalations at a constant pressure of 5 mbar with short breaks in between. A single-use mouthpiece (product No. 100078, ACE Instruments, Germany) was connected to the ionization source, which was heated to 130°C, by a sterilizable, custom-made polytetrafluoroethylene adapter. Measurements were recorded in positive (4500 V) and negative (−4500 V) ionization mode between the *m/z* range of 50–500 Da. The accumulation time was set to 0.5 s per scan. The collisionally activated dissociation (CAD) gas was adjusted to 0 to avoid fragmentation. Temperatures of the MS were set to 0, gas 1 was used to pressurize the vial of the electrospray and set to 24, gas 2 was not connected to the SESI source, and the curtain gas was set to 10. The pulser frequency was adjusted to 23.983 kHz and the pulse 1 duration was 2 µs. A net flow of 0.3 L/min was defined by a mass flow controller (Alicat Scientific, Inc., Tucson, AZ, United States) at the exhaust of the ionization source. The nanoelectrospray was generated using silica emitters (50 cm length, 20 µm diameter, New Objective Inc., Woburn, MA, United States) and a 0.1% (v/v) aqueous formic acid solution (Optima LC/MS Grade, Thermo Fisher Scientific, Waltham, MA, United States). All participants were asked not to brush their teeth, consume any food, drinks (except for water), or chewing gums 1 h prior to the measurements (Weber et al., 2020).

### 2.3 Data preprocessing

All data were recalibrated in PeakView 2.2 (AB Sciex, Concord, ON, Canada) and processed in R version 4.1.1 (R Foundation for Statistical Computing, Vienna, Austria). The conversion and preprocessing of the raw data were done in the same way as described in our previous work (Weber et al., 2020). In brief, the raw mass spectra were resampled by interpolation (Δ*m/z*: 0.0005, *m/z* range: 50–500 Da), peak picking was performed on the average mass spectra associated with exhalation and signal intensities of the *m/z* features were determined by trapezoidal integration. The intensities of the *m/z* features were normalized to the total ion current, log_2_-transformed and arranged into a data matrix of breath profiles for further analysis. More details on data preprocessing are given in the Supplementary Material.

### 2.4 Statistical analysis

To account for confounding influences and reduce the heterogeneity within the groups, batch adjustment was performed by applying surrogate variable analysis (SVA) (Leek and Storey, 2007) on the data matrix of breath profiles. Identification of differentially expressed *m/z* features when comparing cases and controls was assessed by the empirical Bayes moderated t-statistics test (Smyth, 2004). Correction for multiple hypothesis testing was conducted using Benjamini-Hochberg procedure (Benjamini and Hochberg, 1995) with significance threshold set to the adjusted *p*-value of 0.05 to determine the statistically significant features. Additionally, the ability of the breath profiles to classify samples as allergic asthmatic or healthy was assessed with the support vector machines algorithm [13] trained and tested in a 10 times repeated stratified 10-fold cross-validation. To avoid using all features for classifier development, Boruta feature selection (Kursa et al., 2010) was applied in each cross-validation iteration to include only the potentially discriminating features between the allergic asthmatic and the healthy control group. In order to prevent bias during cross-validation all preprocessing steps, feature selection and classifier development were strictly conducted on the training data sets, preventing any information leak from the left-out samples (Varma and Simon, 2006). Details on statistical analysis are given in the supplementary material.

### 2.5 Feature identification

For the 100 most significantly discriminative *m/z* features per study group, compound identification was based on MS^2^ spectra that were recorded directly from exhaled breath by SESI/HRMS with the same instrument set up. The settings of the SESI source and TripleTOF MS were identical to the ones described above for the MS1 full scan acquisition, with the following exceptions: the accumulation time was set to 1.0 s per scan and the CAD gas to 6. Precursors were selected with an isolation window of 0.7 Da. Collision energy for precursor fragmentation was set to 20 eV with a ramped energy spread of ± 10 eV. The MS^2^ spectra were analyzed by a workflow adapted from a published method (Kaeslin et al., 2021) to detect isotopes, adducts, and losses, and with the SIRIUS software (v4.9.9) (Dührkop et al., 2019) to assign putative molecular formulae and chemical structures. The putatively identified compounds were screened for their biological context and subgrouped into metabolic pathways or chemical families. Additionally, pathway enrichment analysis using the mummichog algorithm (MetaboAnalyst, v5.0) (Pang et al., 2021) was performed for further identification on all significant features including those without recorded MS^2^ spectra, without compound suggestions or with excluded MS^2^ spectra (exclusion criteria: see Supplementary Table S1). Lastly, the detected *m/z* features were compared with previously identified compounds from literature. The certainty of identification was indicated by an identification (ID) confidence level ranging from ID 1 to 5, as described by Schymanski and colleagues (Schymanski et al., 2014). More details on the identification approach are included in the supplementary material, including a schematic overview (Supplementary Figure S1).

## 3 Results

### 3.1 Participants and clinical data

Exhaled breath samples of 48 allergic asthma patients and 56 healthy control participants, in total 104 children, were included in this study. The age and body mass index values of the two cohorts were comparable, whereas the asthmatic group contained more males than the healthy one. Detailed clinical characteristics of the two individual study cohorts are shown in Table 1. The use of short-acting beta-agonists was allowed until the day before measurements. All children with asthma had a known allergic sensitization to at least one aero-allergen and the asthma severity ranged from mild to moderate. The FeNO values were significantly elevated in the allergic asthma cohort. Additionally, the forced expiratory volume in 1 s (FEV1) of the asthmatics was lower, while the forced vital capacity of the groups was comparable. Twelve children of the healthy control group showed an allergic sensitization according to the skin prick test, but only two reported symptomatic allergies.

### 3.2 Discriminative breath patterns and their metabolic associations

**FIGURE 1:**
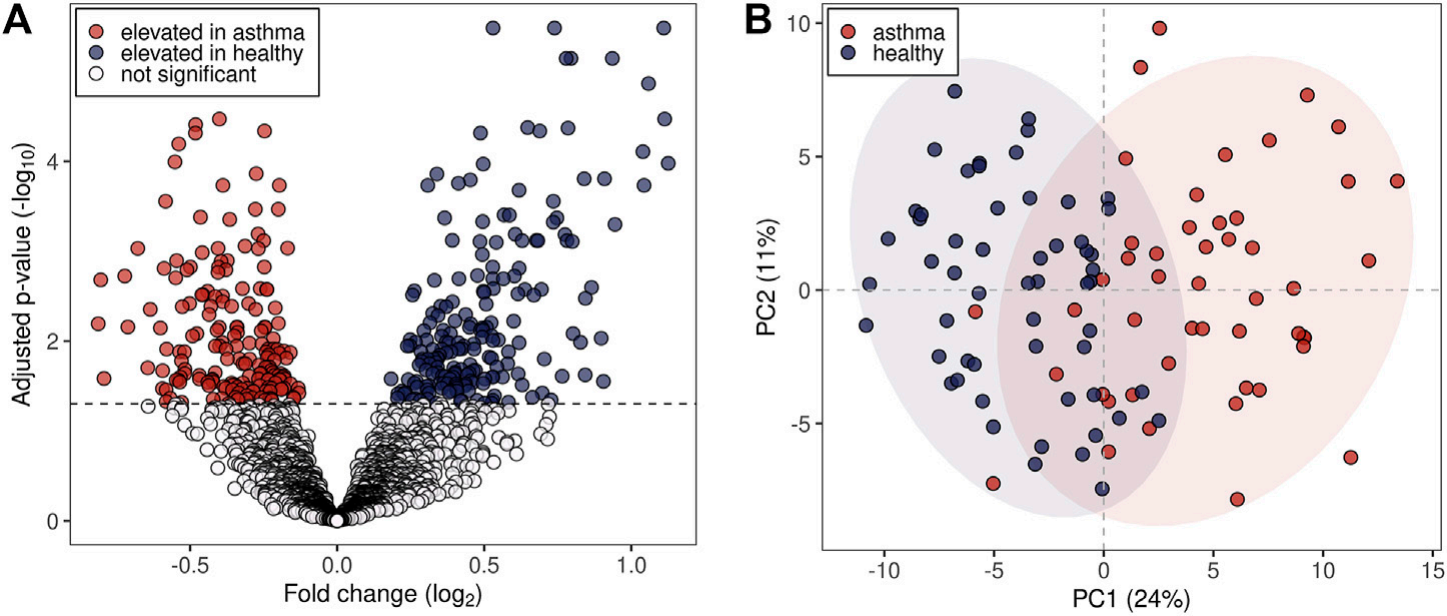
Statistical analysis of *m/z* features in breath profiles. **(A)** Volcano plot representing all detected 2,315 *m/z* features. Dashed line: Benjamini-Hochberg adjusted *p*-value of 0.05. **(B)** First two principal components (PCs) score plot of the 134 putatively identified *m/z* features. Blue dots represent healthy probands and red dots asthmatic patients. 95% data ellipses were added per group for visual depiction.

The pre-processing of the acquired mass spectra of the study subjects revealed 2,315 *m/z* features associated with exhaled breath. 375 *m/z* features were found to be significantly different between the two groups (Benjamini-Hochberg adjusted *p* < 0.05), of which 179 were upregulated and 196 downregulated in the allergic asthma group (Figure 1A). Among those, 134 were assigned to compounds. Inspection of the first two principal components (PCs) of the 134 putatively identified features revealed a moderate separation between the groups along the first PC (24% variance in the data, Figure 1B).

**FIGURE 2:**
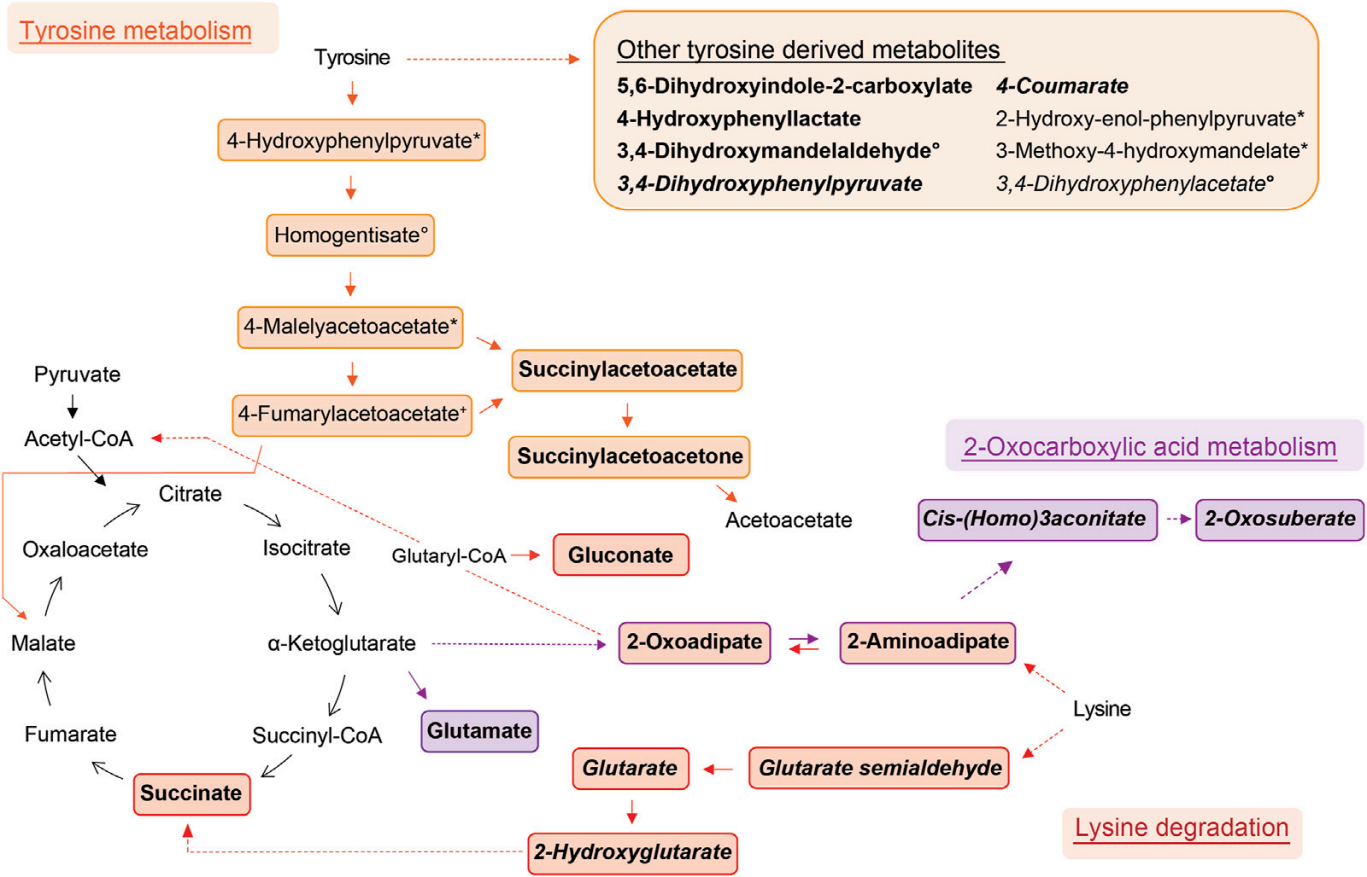
Schemes of metabolic pathways well-represented by compounds that were elevated in the allergic asthma group and putatively identified. Tyrosine derived metabolites besides the main degradation pathway in humans are summarised in the box. Two unrelated compounds of the 2-oxocarboxylic acid metabolism are not shown (see Table 2). Solid lines: direct metabolic relations; dashed lines: indirect metabolic relations (metabolites in between were not identified); colored: putatively identified compound; bold: identified by MS^2^, regular: identified based on exact mass and pathway mapping, or on literature; italic: metabolites from gut microbiota; ^*^, ^°^, ^+^, ^#^, ^, ‘: several possibilities for 1 *m/z* feature based on exact mass and pathway mapping.

**FIGURE 3:**
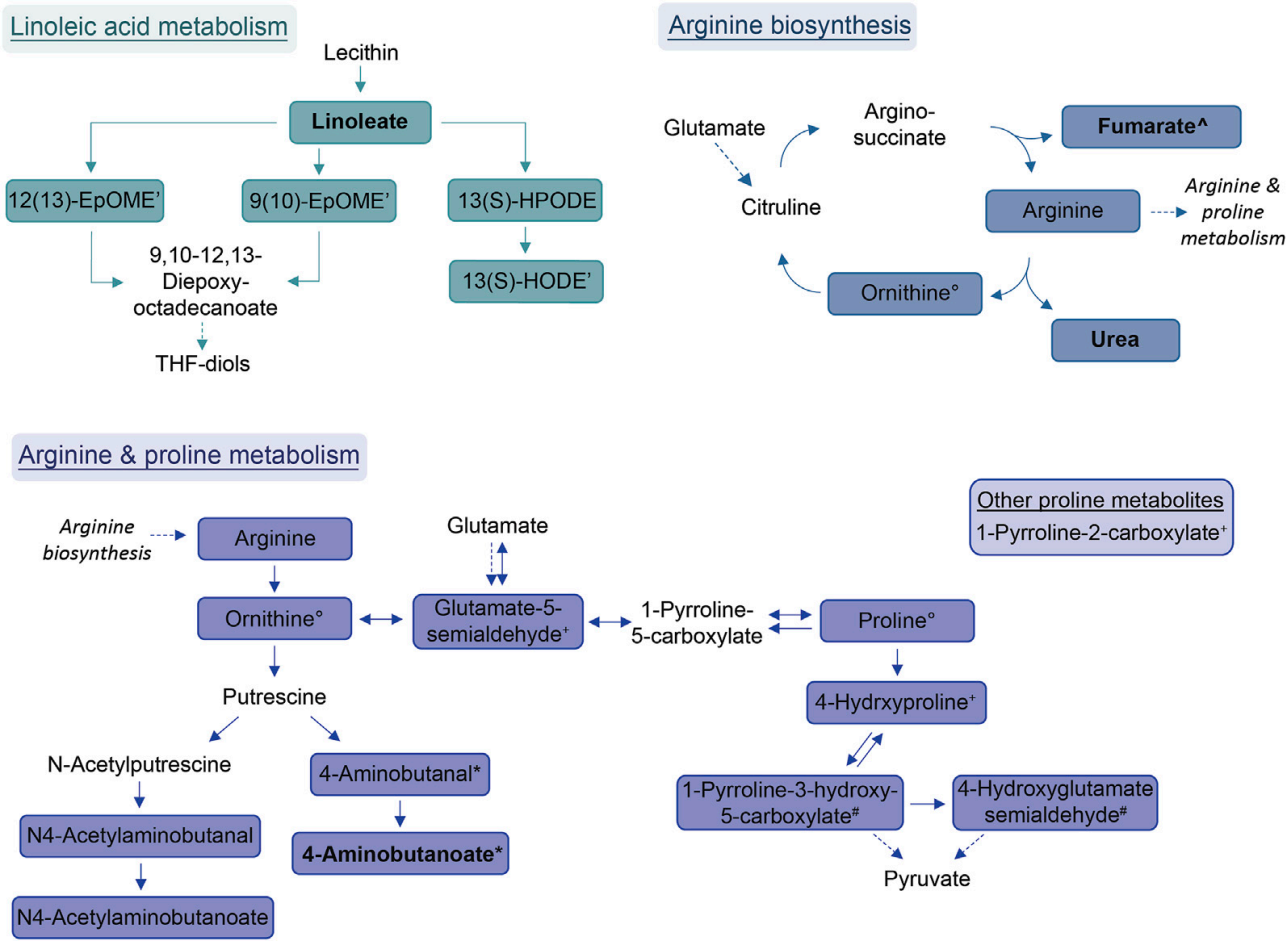
Schemes of metabolic pathways well-represented by compounds that were decreased in the allergic asthma group and putatively identified. Proline metabolism: One component is not directly connected to the displayed pathway and is shown in the box. Solid lines: direct metabolic relations; dashed lines: indirect metabolic relations; colored: putatively identified compound; bold: identified by MS^2^; regular: identified based on exact mass and pathway mapping, or on literature; ^*^, ^°^, ^+^: several possibilities for 1 *m/z* feature based on exact mass and pathway mapping.

Compound identification revealed several specific metabolic pathways and chemical families with many representatives for both study cohorts (Tables 2, 3). For the allergic asthma group, the chemical families of fatty acid metabolites and monosaccharides as well as the 2-oxocarboxylic acid metabolism and two amino acid pathways, i.e., lysine degradation and tyrosine metabolism, were elevated (Table 2). The relations of metabolites involved in some of these elevated pathways are visualized in Figure 2. For the diminished compounds, arginine pathways were found to be well represented, including both arginine and proline metabolism and arginine biosynthesis. Further, several compounds of the linoleic acid metabolism and of the chemical groups of aldehydes, amides, and fatty acids were identified (Table 3; Figure 3). A full list including more details about the putatively identified compounds can be found in Supplementary Table S2.

**TABLE 2:** Pathway related metabolites elevated in the allergic asthma cohort.

*m/z*	Charge	Adj. *p*-value	Log-fold- change	Molecular formula	Ionisation	∆m (ppm)	Compound	Ann.	ID level
**Lysine degradation**									
131.035	neg	0.0002	−0.39	C5H8O4	[M-H]-	0.1	Glutarate (pentanedioic acid)^†^	MS^2^, Lit.	ID1
117.019	neg	0.0004	−0.37	C4H6O4	[M-H]-	−2.8	Succinate (butanedioic acid)^†^	MS^2^, Lit.	ID1
129.019	neg	0.0008	−0.25	C5H8O5	[M-H2O-H]-	−2.6	2-Hydroxyglutarate (2-hydroxypentanedioc acid)	MS^2^	ID3
131.033	pos	0.0031	−0.46	C5H6O4	[M + H]+	−6.8	Glutaconate (2-pentenedioic acid)	MS^2^	ID3
161.0435	pos	0.0032	−0.28	C6H8O5	[M + H]+	−5.9	2-Oxoadipate (2-oxohexanedioic acid)^†^	MS^2^	ID3
162.0755	pos	0.0070	−0.25	C6H11NO4	[M + H]+	−3.6	2-Aminoadipate (2-aminohexanedioic acid)	MS^2^	ID3
97.029	neg	0.0137	−0.20	C5H8O3	[M-H2O-H]-	−5.2	Glutarate semialdehyde (ω-oxopentanoaic acid)	MS^2^	ID3
**Tyrosine metabolism**									
192.0285	neg	0.0001	−0.28	C9H7NO4	[M-H]-	−9.0	5,6-Dihydroxyindole-2-carboxylate	MS^2^	ID3
177.075	pos	0.0017	−0.41	C7H10O4	[M + H2O + H]+	−4.2	Succinylacetone	MS^2^	ID3
183.0295	neg	0.0038	−0.30	C8H10O6	[M-H2O-H]-	−2.2	Succinylacetoacetate	MS^2^	ID3
215.052	pos	0.0076	−0.41	C9H8O5	[M + H2O + H]+	−14.0	3,4-Dihydroxyphenylpyruvate	MS^2^	ID3
181.0505	neg	0.0123	−0.23	C9H10O4	[M-H]-	−0.7	4-Hydroxyphenyllactate	MS^2^	ID3
163.039	neg	0.0134	−0.16	C9H8O3	[M-H]-	−6.6	4-Coumarate	MS^2^	ID3
149.0245	neg	0.0163	−0.21	C8H8O4	[M-H2O-H]-	0.6	3,4-Dihydroxymandelaldehyde	MS^2^	ID3
179.036	neg	0.0214	−0.22	C9H8O4	[M-H]-	5.7	4-Hydroxyphenylpyruvate, 4-Hydroxy-enol-phenylpyruvate	MS^1^	ID4
215.052	neg	0.0268	−0.23	C9H10O5	[M-H2O-H]-	5.7	3-Methoxy-4-hydroxymandelate	MS^1^	ID4
199.025	neg	0.0225	−0.22	C8H8O6	[M-H]-	0.9	4-Maleylacetoacetate, 4-Fumarylacetoacetate	MS^1^	ID4
197.046	neg	0.0268	−0.23	C9H10O5	[M-H]-	2.3	3-Methoxy-4-hydroxymandelate^†^	MS^1^	ID4
167.0345	neg	0.0271	−0.19	C8H8O4	[M-H]-	−2.9	Homogentisate, 3,4-Dihydroxymandelaldehyde, 3,4-Dihydroxyphenylacetate	MS^1^	ID4
**2-Oxocarboxylic acid metabolism**									
169.05	neg	0.0006	−0.27	C8H12O5	[M-H2O-H]-	−3.7	2-Oxosuberate (2-oxooctanedionic acid)	MS^2^	ID3
199.058	pos	0.0010	−0.46	C9H12O6	[M-H2O + H]+	−10.5	cis-(Homo)3-aconitate	MS^2^	ID3
159.0645	pos	0.0015	−0.40	C7H12O5	[M-H2O + H]+	−4.3	3-Isopropylmalate	MS^2^	ID3
161.0435	pos	0.0032	−0.28	C6H8O5	[M + H]+	−5.9	2-Oxoadipate (2-oxohexanedioic acid)^†^	MS^2^	ID3
162.0755	pos	0.0070	−0.25	C6H11NO4	[M + H]+	−3.6	2-Aminoadipate (2-aminohexanedioic acid)	MS^2^	ID3
148.06	pos	0.0077	−0.26	C5H9NO4	[M + H]+	−2.9	Glutamate	MS^2^	ID3
146.0545	neg	0.0404	−0.31	C5(13C)H10O4	[M(C13)-H]-	7.3	2-Aceto-2-hydroxybutanoate	MS^1^	ID4
**Fatty acid metabolites**									
117.019	neg	0.0004	−0.37	C4H6O4	[M-H]-	−2.8	Butanedioic acid (succinate)^†^	MS^2^, Lit.	ID1
131.035	neg	0.0002	−0.39	C5H8O4	[M-H]-	0.1	Pentanedioic acid (glutarate)^†^	MS^2^, Lit.	ID1
147.0645	pos	0.0070	−0.40	C6H10O4	[M + H]+	−4.7	Hexanedioic acid (adipic acid)	MS^2^	ID3
131.033	pos	0.0031	−0.46	C5H6O4	[M + H]+	−6.8	Pentenedioic acid (glutaconate)	MS^2^	ID3
143.0345	neg	0.0363	−0.23	C6H8O4	[M-H]-	−3.7	Hexenedioic acid	Lit.	ID4
157.0505	neg	0.0230	−0.26	C7H10O4	[M-H]-	−0.8	Heptenedioic acid^†^	Lit.	ID4
97.029	neg	0.0137	−0.20	C5H8O3	[M-H2O-H]-	−5.2	ω-Oxopentanoaic acid (glutarate semialdehyde)	MS^2^	ID3
125.06	neg	0.0195	−0.23	C7H12O3	[M-H2O-H]-	−6.4	ω-Oxoheptanoic acid	MS^2^	ID3
113.024	neg	0.0424	−0.15	C5H8O4	[M-H]-	−3.7	ω-Oxopentenoic acid	Lit.	ID4
155.071	neg	0.0268	−0.20	C8H12O3	[M-H]-	−2.1	ω-Oxooctenoic acid	Lit.	ID4
167.071	neg	0.0264	−0.18	C9H12O3	[M-H]-	−1.9	ω-Oxononadienoic acid	Lit.	ID4
181.086	neg	4.83E-05	−0.48	C10H14O3	[M-H]-	−5.6	ω-Oxodecadienoic acid	Lit.	ID4
87.0445	neg	0.0471	−0.58	C4H8O2	[M-H]-	−7.5	Butanoic acid^†^	Lit.	ID2
101.0605	neg	0.0214	−0.59	C5H10O2	[M-H]-	−0.2	Pentanoic acid^†^	Lit.	ID2
197.081	neg	0.0004	−0.46	C10H14O4	[M-H]-	−4.6	2,7-Dimethyl-2,4-octadienedioic acid	MS^2^	ID3
129.019	neg	0.0008	−0.25	C5H8O5	[M-H2O-H]-	−2.6	2-Hydroxypentanedioc acid (2-hydroxyglutarate)	MS^2^	ID3
178.0355	neg	0.0129	−0.18	C5H9NO6	[M-H]-	−1.2	2-Amino-3,4-dihydroxypentanedioic acid	MS^2^	ID3
161.0435	pos	0.0032	−0.28	C6H8O5	[M + H]+	−5.9	2-Oxohexanedioic acid (2-oxoadipate) ^†^	MS^2^	ID3
162.0755	pos	0.0070	−0.25	C6H11NO4	[M + H]+	−3.6	2-Aminohexanedioic acid (2-aminoadipate)	MS^2^	ID3
133.05	neg	0.0104	−0.39	C5H10O4	[M-H]-	−4.8	2,3-Dihydroxypentanoic acid	MS^2^	ID3
**Monosaccharides and metabolites**									
163.024	neg	0.0002	−0.20	C5H8O6	[M-H]-	−5.0	2-Dehydro-xylonate	MS^2^	ID3
151.0585	pos	0.0013	−0.51	C5H10O5	[M + H]+	−10.6	Arabinose	MS^2^	ID3
163.0595	pos	0.0158	−0.33	C6H12O6	[M-H2O + H]+	−3.7	Galactose^†^	MS^2^	ID3
193.035	neg	0.0196	−0.24	C6H10O7	[M-H]-	−2.0	Glucuronate	MS^2^	ID3
209.03	neg	0.0244	−0.20	C6H10O8	[M-H]-	−1.4	Glucarate	MS^1^	ID4
91.04	neg	0.0261	−0.79	C3H8O3	[M-H]-	−0.7	Glycerol	MS^1^	ID4
119.0345	neg	0.0319	−0.39	C4H8O4	[M-H]-	−4.1	Erythrulose	MS^1^	ID4

Putatively identified compounds elevated in the allergic asthma cohort, grouped by metabolic pathways or chemical families and ordered by their adjusted *p*-value. Exception: fatty acid metabolites are sorted based on their chemical relation. Log-fold-change: negative values indicate higher average expression in the asthmatic group. Log-fold-change was calculated using R-package “limma” (Ritchie et al., 2015); see Supplementary Section S3 for more details. The listed *m/z* values represent the measured values and the mass error (∆m) to the theoretical mass is reported in ppm. Annotation (Ann.) e.g., based on literature (Lit.), references for literature-based identification are included in Supplementary Table S2. †: compounds that were detected several times in different ionisation forms (listed in Supplementary Table S2). MS^1^: assignment based on full scan mode by literature match or pathway analysis, MS^2^: assignment based on real-time tandem mass spectrometry spectra, ID: identification confidence level ranging from ID1 (high) to ID5 (low).

**TABLE 3:** Pathway related metabolites downregulated in the allergic asthma cohort.

*m/z*	Charge	Adj. *p*-value	Log-fold-change	Molecular formula	Ionisation	∆m (ppm)	Compound	Ann.	ID level
**Arginine and proline metabolism**									
104.07	pos	0.0002	0.41	C4H9NO2	[M + H]+	−5.8	4-Aminobutanoate^†^	MS^2^, Lit.	ID3
60.0805	pos	0.0083	0.80	C4H9NO	[M-CO + H]+	−4.7	4-Aminobutanal	MS^1^	ID4
				C4H9NO2	[M-CO2+H]+	−4.7	4-Aminobutanoate	MS^1^	ID4
116.07	pos	0.0122	0.42	C5H9NO2	[M + H]+	−5.2	Proline	MS^1^, Lit.	ID4
				C5H12N2O2	[M-NH3+H]+	−5.2	Ornithine	MS^1^	ID4
193.13	pos	0.0251	0.53	C6H14N4O2	[M + H2O + H]+	2.5	Arginine	MS^1^	ID4
118.086	pos	0.0252	0.39	C6H11NO3	[M-CO + H]+	−2.2	4-Acetamidobutanoate	MS^1^	ID4
114.0545	pos	0.0264	0.38	C5H7NO2	[M + H]+	−4.0	1-Pyrroline-2-carboxylate^†^	MS^1^	ID4
				C5H9NO3	[M-H2O + H]+	−4.0	Hydroxyproline, Glutamate 5-semialdehyde	MS^1^	ID4
112.075	pos	0.0313	0.31	C6H11NO2	[M-H2O + H]+	−6.2	N4-Acetylaminobutanal	MS^1^	ID4
102.0545	pos	0.0381	0.31	C5H9NO4	[M-HCOOH + H]+	−4.5	4-Hydroxyglutamate semialdehyde	MS^1^	ID4
				C5H7NO3	[M-CO + H]+	−4.5	1-Pyrroline-3-hydroxy-5-carboxylate^†^	MS^1^	ID4
**Arginine biosynthesis**									
61.039	pos	0.0076	0.36	CH4N2O	[M + H]+	−10.5	Urea	MS^2^	ID3
96.9925	neg	0.0113	0.25	C4H4O4	[M-H2O-H]-	−6.4	Fumarate	MS^2^	ID3
				C4H4O4	[M-H2O-H]-	−6.4	Maleate	MS^2^	ID3
116.07	pos	0.0122	0.42	C5H9NO2	[M + H]+	−5.2	Proline	MS^1^, Lit.	ID4
				C5H12N2O2	[M-NH3+H]+	−5.2	Ornithine	MS^1^	ID4
193.13	pos	0.0251	0.53	C6H14N4O2	[M + H2O + H]+	2.5	Arginine	MS^1^	ID4
102.0545	pos	0.0381	0.31	C5H9NO4	[M-HCOOH + H]+	−4.5	4-Hydroxyglutamate semialdehyde	MS^1^	ID4
				C5H7NO3	[M-CO + H]+	−4.5	1-Pyrroline-3-hydroxy-5-carboxylate	MS^1^	ID4
**Linoleic acid metabolism**									
281.2475	pos	7.12E-06	0.79	C18H32O2	[M + H]+	−0.04	Linoleate^†^	MS^2^	ID3
295.225	pos	2.09E-04	0.62	C18H32O4	[M-H2O + H]+	−6.0	13(S)-HPODE^†^	MS^1^	ID4
297.242	pos	4.73E-04	0.73	C18H32O3	[M + H]+	−1.4	13(S)-HODE^†^, 12 (13)-EpOME^†^, 9 (10)-EpOME^†^	MS^1^	ID4
**Aldehydes**									
115.075	pos	0.0491	0.49	C6H10O2	[M + H]+	−3.1	4-Hydroxy-2-hexenal^†^	Lit.	ID2
146.117	pos	0.0210	0.30	C7H12O2	[M + NH4]+	−3.8	4-Hydroxy-2-heptenal	Lit.	ID4
143.106	pos	0.0179	0.41	C8H14O2	[M + H]+	−4.6	4-Hydroxy-2-octenal^†^	Lit.	ID4
258.243	pos	0.0225	0.34	C15H28O2	[M + NH4]+	1.0	4-Hydroxy-2-pentadecenal	Lit.	ID4
158.1175	pos	0.0082	0.39	C8H12O2	[M + NH4]+	−0.4	4-Hydroxy-2,6-octadienal	Lit.	ID4
172.133	pos	0.0004	0.57	C9H14O2	[M + NH4]+	−1.2	4-Hydroxy-2,6-nonadienal	Lit.	ID2
228.196	pos	0.0238	0.44	C13H22O2	[M + NH4]+	0.9	4-Hydroxy-2,6-tridecadienal	Lit.	ID2
283.191	neg	0.0292	0.46	C15H26O2	[M + HCOO]-	−1.7	4-Hydroxy-2,6-pentadecadienal	Lit.	ID4
253.2155	pos	0.0008	0.49	C16H28O2	[M + H]+	−2.8	4-Hydroxy-2,6-hexadecadienal	Lit.	ID4
**Fatty amides**									
200.201	pos	0.0008	0.63	C12H25NO	[M + H]+	0.5	Dodecanamide	MS^2^	ID3
256.263	pos	0.0008	0.80	C16H33NO	[M + H]+	−1.9	Hexadecanamide	MS^2^	ID3
302.305	pos	0.0093	0.90	C18H37NO	[M + H2O + H]+	−1.2	Octadecanamide	MS^2^	ID3
288.253	pos	0.0003	0.74	C16H33NO3	[M + H]+	−1.1	N,N-bis(2-hydroxyethyl)dodecanamide	MS^2^	ID3
316.2845	pos	0.0001	1.04	C18H35NO2	[M + H2O + H]+	−0.4	Palmitoleoylethanolaimde	MS^2^	ID3
318.3	pos	0.0001	1.13	C18H37NO2	[M + H2O + H]+	−0.8	Palmitoylethanolamide	MS^2^	ID3
**Fatty acids**									
271.2265	pos	0.0006	0.77	C16H32O4	[M-H2O + H]+	−1.0	10,16-Dihydroxyhexadecanoic acid	MS^2^	ID3
220.1905	pos	0.0041	0.37	C11H23NO2	[M + H2O + H]+	−1.0	11-Aminoundecanoic acid	MS^2^	ID3
151.096	pos	0.0159	0.29	C6H12O3	[M + H2O + H]+	−3.2	6-Hydroxyhexanoic acid	MS^1^	ID4

Putatively identified compounds downregulated on the allergic asthma cohort grouped by metabolic pathways or chemical families and ordered by their adjusted *p*-value. Exception: aldehydes and fatty amides are sorted based on their chemical relation. Log-fold-change: positive values indicate higher average expression in the healthy group. Log-fold-change was calculated using R-packege “limma” (Ritchie et al., 2015); see supplementary material section S3 for more details. The listed *m/z* values represent the measured values and the mass error (∆m) to the theoretical mass is reported in ppm Annotation (Ann.) e.g., based on literature (Lit.), references for literature-based identification are included in Supplementary Table S2. †: compounds that were detected several times in different ionisation forms (listed in Supplementary Table S2). MS^1^: assignment based on full scan mode by literature match or pathway analysis, MS^2^: assignment based on real-time tandem mass spectrometry spectra, ID: identification confidence level ranging from ID1 (high) to ID5 (low). 12(13)-EpOME: 12,13-Epoxyoctadec-9(Z)-enoic acid; 9 (10)-EpOME: 9,10-Epoxyoctadec-12(Z)-enoic acid; 13(S)-HPODE: 13(S)-Hydroperoxy-9Z, 11E-octadecadienoic acid; 13(S)-HODE: 13(S)-Hydroxy-9Z, 11E-octadeca-dienoic acid.

**FIGURE 4:**
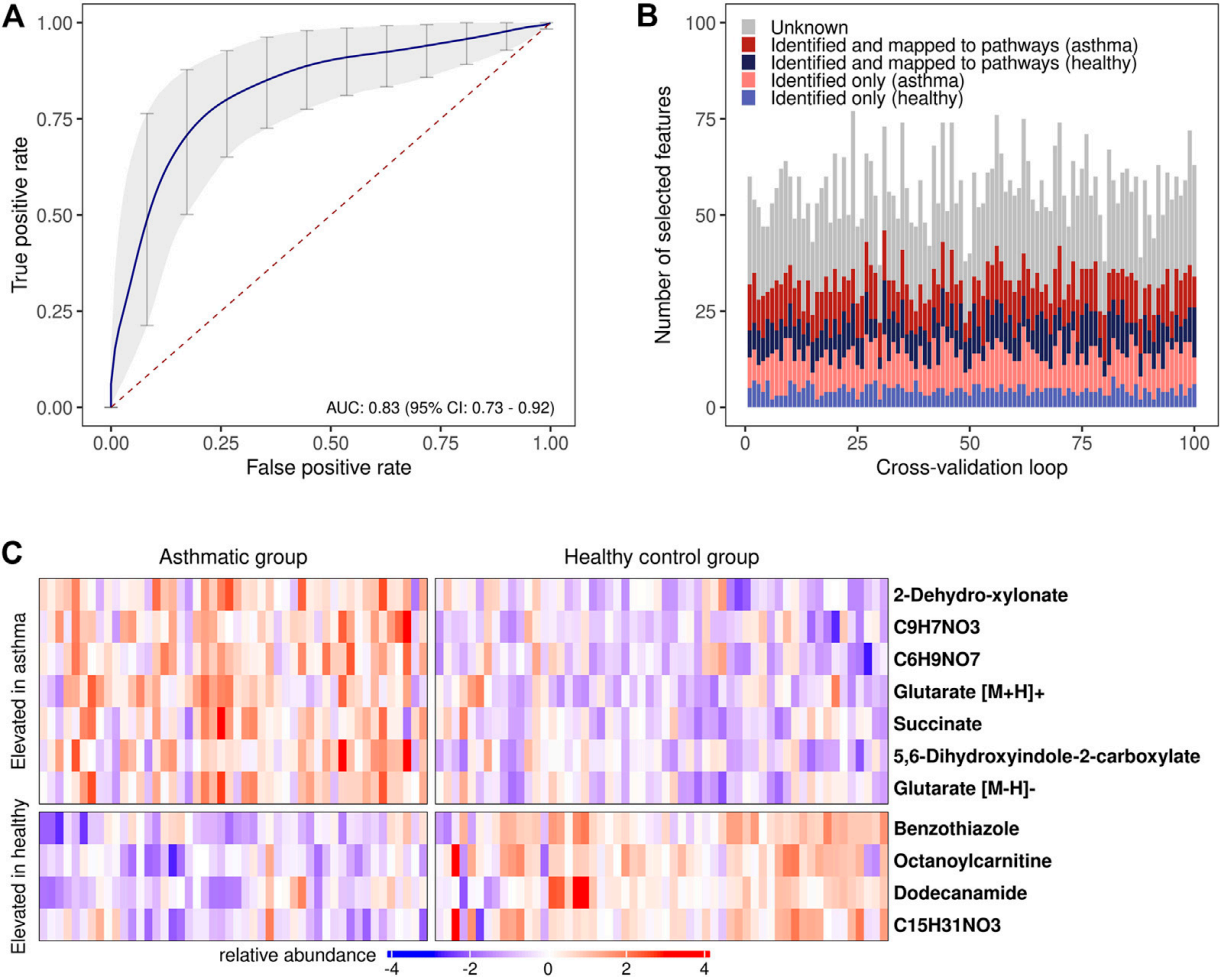
Disease prediction based on breath profiles. **(A)** Average receiver operating characteristic curve (ROC) with an average AUC of 0.83 resulting from the 10 times repeated 10-fold cross-validation. ROC curves resulting from predictions on each of the left-out data sets in the cross-validation were used to calculate the average ROC curve (Fawcett, 2006) (vertical averaging). Vertical grey bars: pointwise confidence intervals computed using bootstrapping (10.000 repetitions); red dashed line: line of no discrimination. **(B)** Stacked bar plots of the selected features in each cross-validation iteration. Red/blue color scheme: upregulated features in the allergic asthmatic/healthy group. **(C)** Heat map of the most frequently chosen features (standardized intensities) as predictors in the cross-validation. Columns: study participants; rows: *m/z* features with putatively identified compounds (right), a chemical formula is provided if compound identification was not possible.

The assessment of the classification accuracy in discriminating between the allergic asthmatic and the healthy samples resulted in an area under the curve (AUC) of 0.83, 95% CI: 0.73–0.92, (Figure 4A; Supplementary Table S3; Supplementary Figure S2). When examining feature selection by the Boruta scheme (Kursa et al., 2010) within cross-validation, 57 (±8) *m/z* features were selected on average in each cross-validation iteration, many of which were putatively identified with the compound identification workflow above (Figure 4B). Compounds which were most frequently selected in LOOCV are presented in Figure 4C (for box plots see Supplementary Figure S2) and all the other selected metabolites can be found in Supplementary Table S4.

It is of relevance to note that the adjustment with SVA captures the components of variability within the data and reduces any effect on the intensity levels of *m/z* features arising from other sources than the primary variables of interest (i.e., allergic asthma vs. healthy controls). Hence, any further subgroup analysis or correlation analysis to other clinical parameters could not be performed (Leek and Storey, 2007) Nevertheless, we decided to assess whether atopy by itself has an impact on the breath profiles by isolating the group of healthy samples and repeating our analysis pipeline to find differences between the healthy children with sensitization and the ones without. We found no significantly different features between the two groups (see Supplementary Figure S5).

## 4 Discussion

We present the first online breath analysis study performed by SESI/HRMS on a pediatric population with allergic asthma. The study revealed group-specific breath patterns with a large number of discriminative *m/z* features, many of which were putatively identified and could be grouped to metabolic pathways or chemical families. Moreover, some of the relevant compounds and pathways were previously published in metabolomic studies in pediatric asthma (Neerincx et al., 2017; Ferraro et al., 2018; Papamichael et al., 2021) or reported in SESI/HRMS studies (see Supplementary Table S2).

As described by Papamichael et al., an altered energy metabolism is expected in children with asthma due to the hypoxic environment, bronchoconstriction, and other associated changes as well as increased efforts for breathing (Papamichael et al., 2021). However, this explanation might not apply to the included asthmatic group of our study, as they did not suffer from acute exacerbations. The lung and gut microbiomes are also potential contributors to the pathophysiology of asthma (Barcik et al., 2020). Several previous breath analysis studies identified compounds and molecular pathways associated with pediatric asthma. However, some of the potential biomarkers were of exogenous origin and only a handful of them were consistently detected in more than one study (Neerincx et al., 2017; Ferraro et al., 2018; Papamichael et al., 2021). The pathways and chemical families identified in our study are biologically relevant and reflect both an altered state of energy metabolism as well as changes in products from the microbiome.

The metabolism of lysine was the most significantly elevated pathway in asthma and all associated compounds were identified based on direct MS^2^ spectra. Two different degradation pathways of lysine were found, one is taking place in humans and the other in the gut microbiota (Figure 2). The associated metabolites succinate and glutarate were unambiguously identified (ID1, see Table 2), and have been reported as associated with pediatric asthma in previous metabolomic studies in blood (Chang et al., 2015), urine (Saude et al., 2011), and breath (Carraro et al., 2018). Carraro et al. also reported a decreased level of oxoadipate in early asthma, which is not in line with our findings but could be explained by the different study design focusing on wheezing in preschool children (Carraro et al., 2018). However, a study linked an enzymatic complex involved in the lysine degradation pathway to the formation of reactive oxygen species from 2-oxoadipate (Jordan et al., 2019), which could potentially be a link to asthma pathophysiology.

Tyrosine metabolism was also significantly upregulated in the allergic asthmatic group. As illustrated in Figure 2, some of the metabolites belong to the main human degradation pathway whereas other tyrosine-derived metabolites are of human or microbiotic origin. An increased level of tyrosine in asthmatic children was reported in previous metabolomics studies (Saude et al., 2011; Papamichael et al., 2019; Tao et al., 2019). Additionally, the bacterial tyrosine metabolite 4-hydroxyphenylacetate was reported to be negatively correlated with the FEV1 in urine (Papamichael et al., 2019). It is hypothesized that high levels of tyrosine metabolism might be related to inflammation and oxidative stress in asthma (Papamichael et al., 2021). Also, tyrosine-derived catecholamines are important during conditions of stress and play a role in the regulation of the immune system (Barnes et al., 2015). In contrast to these findings, Carraro et al. reported a lower level of some tyrosine metabolites in children with early asthma compared to transient wheezers (Carraro et al., 2018).

The largest elevated group consisted of 20 fatty acid metabolites, including saturated and unsaturated dicarboxylic acids, *ω*-oxo-acids, hydroxy-acids, and alkanoic acids. All lysine metabolites are additionally fitting into this chemical family. A large part of these identified fatty acids were previously reported being decreased in chronic obstructive pulmonary disease exacerbations by SESI/HRMS and described as metabolites of the *ω*-oxidation, a minor pathway of the fatty acid oxidation (Gaugg et al., 2017a; 2019). Interestingly, important molecules in asthma pathophysiology including arachidonic acid, leukotrienes, and prostaglandins, although not detected in this study, are also common substrates of the cytochrome P450 *ω*-hydroxylases (Ni and Liu, 2021). Therefore, our findings support the hypothesis that *ω*-oxidation might be upregulated in allergic asthma. Butanoic and pentanoic acid are both identified (ID2, Supplementary Table S2) and were both reported to distinguish asthmatic from healthy children in previous studies (Dallinga et al., 2010; van Mastrigt et al., 2016; van Vliet et al., 2016). Furthermore, the findings from previous metabolomic studies of an altered fatty acid metabolism in asthma is supported by our data (Neerincx et al., 2017; Ferraro et al., 2018; Papamichael et al., 2021).

Further, the 2-oxocarboxylic acid metabolism was also elevated in the allergic asthmatic (Figure 2). 2-Oxoadipate and 2-aminoadipate are overlapping with the lysine degradation pathway and glutamate is a common metabolite involved in multiple metabolic pathways. The metabolism of 2-oxocarboxylic acids is solely happening in archaea, which are also represented in the gut microbiota. A review linked methanogenic archaea as potential important contributors to atopic diseases (Sereme et al., 2019). Additionally, 2-oxoadipate was previously detected as an exhaled metabolite from the gut microbiota in a mice model study (Lan et al., 2022).

Lastly, monosaccharides and derived metabolites were increased in allergic asthma. This difference in carbohydrate metabolism of asthmatic children is expected due to an altered energy demand and metabolism (Papamichael et al., 2021).

A recently published study comparing children with acute asthma exacerbations and healthy controls reported similar results to ours. Despite investigating urine by high-performance liquid chromatography mass spectrometry, they also reported an elevated level of tyrosine metabolism including gentisate and increased glucuronate as well as a downregulated linoleic acid metabolite and palmitic acid in children with acute asthma (Li et al., 2022).

The most prominent group of downregulated metabolites was associated with arginine and proline metabolism as well as arginine biosynthesis. Arginase, an enzyme that converts arginine into ornithine and urea is an important contributor to asthma pathophysiology (Maarsingh et al., 2011). According to Maarsingh et al., increased expression and activity of arginase in asthma mouse models resulted in the promotion of inflammatory processes, decreased arginine and increased ornithine and proline levels (Maarsingh et al., 2011). This is not completely in line with our findings where these downstream pathways are decreased in allergic asthma. However, a study about amino acids in blood serum of asthmatics reported decreased arginine, proline and ornithine levels (Morris et al., 2004).

Further, the linoleic acid metabolism was well-represented amongst the diminished compounds. While conjugated linoleic acid was consistently reported as having anti-inflammatory properties, the effect of linoleic acid especially on asthma is in dispute due to controversial observations in clinical trials (Wendell et al., 2014). Interestingly, a more recent study found genetically predicted linoleic acid to be associated with a lower risk for asthma, which is in line with our results (Zhao and Schooling, 2019).

Within the group of amides, palmitoylethanolamide (PEA) was found to be decreased in the allergic asthma group. This is in line with the well-studied anti-inflammatory effect of PEA (Clayton et al., 2021). More recently, also an inhibitory effect for the development of allergic airway symptoms was reported for PEA in mice (Roviezzo et al., 2017).

Another group of downregulated compounds was assigned to aldehydes. Aldehydes are indicative of oxidative stress and originate from lipid peroxidation (Jesenak et al., 2017), which is involved in asthma pathophysiology, and are thus expected to be increased in asthmatics. However, the results in literature on breath analysis in asthma are not consistent: Some studies observed an increased level of certain aldehydes in the asthmatic group (Gahleitner et al., 2013; van de Kant et al., 2013; Smolinska et al., 2014), whereas others reported unaltered or even decreased levels (Ibrahim et al., 2011; Sagdic et al., 2011; Caldeira et al., 2012; Riscassi et al., 2022). Beyond this, aldehydes are also used as common additives in cosmetics or food and are known environmental contaminants (Sinharoy et al., 2019), which could influence their exhaled concentrations. Furthermore, the annotation of aldehydes in our study was based solely on exact mass matches with previously published compounds by SESI/HRMS (see Supplementary Table S2).

Altogether, many of the enriched pathways that we reported either elevated or decreased in allergic asthma could be linked to previous findings of metabolomic studies using various methods for blood, urine, or breath analysis. This strengthens the putative compound identification performed in this work and supports the possible biological and diagnostic value of these metabolites.

Assessing the predictability of the disease with supervised machine learning in a 10 times repeated 10-fold cross-validation revealed an AUC of 0.83 (CI: 0.73–0.92), indicating that the metabolic profiles could be applied for potential diagnostic purposes. Some compounds that were allocated to subgroups of metabolic pathways or chemical families were frequently selected during cross-validation (Figure 4C; Supplementary Table S4) suggesting that a smaller group of compounds might not only be pathophysiologically relevant, but also has potential for diagnostic models. The two dicarboxylic acids and lysine metabolites, succinate and glutarate, are promising candidates and were unambiguously identified. Nevertheless, while efforts have been taken to prevent bias by preprocessing data in each cross-validation loop and reducing the dimensionality of the feature set for training the classifier with machine learning, the risk of overfitting cannot be completely ruled out (Vabalas et al., 2019). An independent and increased study cohort would be needed to help in validating the model performance and the selected predictors (Fijten et al., 2017).

Due to a rather large number of significant *m/z* features, a main focus was set on putative compound identification. We aimed at establishing an objective workflow that is based on matching direct MS^2^ spectra with database fragment spectra, adapted from previous work (Kaeslin et al., 2021), refined for a more extensive screening of suggested compounds, and expanded by pathway enrichment analysis to strengthen the feature annotation. A limitation in our identification approach is the lacking chromatographic separation in SESI/HRMS, which hinders the distinction of isomeric compounds. Further, with a minimum isolation window of 0.7 Da, co-fragmentation of several compounds with similar masses can occur, which complicates the annotation of fragment spectra. To address this, we excluded several MS^2^ spectra with insufficient quality from further analysis, as specified in Supplementary Table S1. The confidence of identification is only moderate for most compounds, as they are putatively annotated based on fragment spectra analysis with the SIRIUS software that uses computational power to determine chemical structures that potentially have a matching fragmentation pattern (Dührkop et al., 2019) or the exact mass comparison to the literature and/or known metabolites. Therefore, there is a general risk for misclassification, and the confirmation of the unambiguous chemical structures requires further time-consuming experiments, including the measurement of standards. Especially, the molecules that were annotated based on their exact mass or solely detected as adduct or loss species without their primary ion being amongst the significant features need further investigation. However, our aim for this study was to get a broad overview over the potentially involved metabolic pathways rather than accurately identifying a small set of single compounds.

A strength of this study design is that all enrolled patients were taken off long-term therapy at least 1 week prior to the study visit and did not take any short-acting relievers on the day of measurement. While direct breath analysis by SESI bypasses any contamination during sample preparation, this adds up to also diminish confounders and signal interferences from medications in exhaled breath. This is an important aspect, as it was previously shown that the methodology can detect drugs, including the asthma medication Salbutamol, in breath (Gaugg et al., 2017b; Chen et al., 2021; Singh et al., 2021).

While asthma is a heterogeneous disease with different phenotypes, this study focused only on allergic asthma, the most frequent phenotype in children. Therefore, our findings cannot be extrapolated to all forms of pediatric asthma. We included all sensitized healthy controls and all asthmatics with allergic comorbidities such as allergic eczema or hay fever in order to represent the real population for future applications. 21.4% of the healthy cohort had an allergic sensitization to at least one common aeroallergen, which is in line with the estimated prevalence in children (Kölli et al., 2022). However, performing subgroup analysis on the entire data set was not feasible since surrogate variable analysis is known to adjust for confounding influences and reduce heterogeneity in the data (e.g., demographic variations like age and sex, or disease heterogeneity) (Jaffe et al., 2015). We decided to apply SVA to adjust for unmodeled factors, since the study was conducted over a period of 15 months on a highly sensitive instrument. Therefore, despite a strict adherence to standard operating procedures, we had to assume that apart from demographic variation also unknown environmental or technical confounders might have impacted the *m/z* feature intensities. In order to nevertheless assess the interesting question whether the described markers and pathways might also be related to atopy by itself, we chose to perform an independent subgroup analysis in the healthy cohort. No significant features that could distinguish healthy children with allergic sensitization from the ones without could be found. Therefore, the identified metabolites and pathways represent promising candidate biomarkers for allergic asthma that need to be validated in a larger and independent study cohort.

This study confirms the applicability of SESI/HRMS to a pediatric population and shows its potential to distinguish children with allergic asthma from healthy controls based on their breath signatures. Moreover, well-represented metabolic pathways that are potentially linked to the pathophysiology of allergic asthma in children could be identified. A smaller subset of the differentiating compounds could possibly be used for predictive modelling. These findings might set the path for much-needed, non-invasive clinical applications to improve early diagnosis of asthma.

## Data Availability

TThe MS^2^ data generated for this study can be found in the FigShare repository at doi: 10.6084/m9.figshare.21946877. Due to ethical restrictions, other data is available upon request from the authors.
