# Supplementary material for: Online breath analysis with SESI/HRMS for metabolic signatures in children with allergic asthma
Source: Front Mol Biosci. 2023 Mar 31;10:1154536. doi: 10.3389/fmolb.2023.1154536 (PMC10102578; doi:10.3389/fmolb.2023.1154536)
Supplement: Supplementary file 2 [file DataSheet1.docx]

Supplementary Material

Online Breath Analysis by SESI/HRMS for Metabolic Signatures in Children with Allergic Asthma

Ronja Weber^1†^, Bettina Streckenbach^2†^, Lara Welti^1^, Demet Inci^1^, Malcolm Kohler^3^, Nathan Perkins^4^, Renato Zenobi^2^, Srdjan Micic^1‡^, Alexander Moeller^1‡*^

^1^Department of Respiratory Medicine, University Children's Hospital Zurich, Zurich, Switzerland

^2^Department of Chemistry and Applied Biosciences, ETH, Zurich, Zurich, Switzerland

^3^Department of Pulmonology, University Hospital Zurich, Zurich, Switzerland ^4^Division of Clinical Chemistry and Biochemistry, University Children's Hospital Zurich, Zurich, Switzerland

^†^These authors contributed equally to this work and share first authorship

^‡^These authors contributed equally to this work and share last authorship

*** Correspondence:** Alexander Moeller
alexander.moeller@kispi.uzh.ch

# S1 Participants and clinical data

Information about this observational study is provided according to the STROBE guidelines. A total of 179 participants, 97 with allergic asthma and 82 children without asthma were contacted for participation. 29 out of 179 either declined to participate, did not show up or could not stop their medication (in case of asthma patients). From the 72 patients and 78 healthy children who entered the study, seven patients were excluded due to errors in recruitment and 39 participants (26%) had to be excluded during the measurements for not being able to properly conduct the breath maneuvers or due to technical problems with the instrumental setup. The data of 104 participants, 48 allergic asthmatics and 56 healthy controls, was included in the final study.

For all participants, anthropometric data including sex, height and weight was collected. For the healthy control group, children without any diagnosed lung diseases were recruited via personal connections, flyers and advertisements on social media. Participants of the control cohort performed additional clinical tests during the study visit to exclude undiagnosed lung diseases and detect allergic sensibilizations. The tests included a spirometry (SpironSensePro 1.3, PARI GmbH, Germany) and an offline quantitation of fractional exhaled nitric oxide (FeNO) with a Quintron bag (QuinTron Instrument Company, Inc., Milwaukee, WI, USA). The FeNO concentration was analyzed by Spiroware® Software (v. 3.2.1, Eco Medics AG, Dürnten, Switzerland). Further, the control subjects were screened for sensibilizations for five common air-borne allergens using a skin prick test (Soluprick, ALK-Abello Ltd., Berkshire, UK). The test included screening for grass mixture, birch, hasel, house dust mite and cat hair as well as a positive (histamine) and negative control. Details about respiratory health and family history of the control subjects were additionally obtained by a questionnaire (LuftiBus®, Verein Lunge Zürich, Switzerland).

For asthmatic children, the clinical data was retrieved from their clinical records. Spirometry and online FeNO quantitation for asthmatic participants were performed on the same day as their breath analysis measurement as part of their routine visit at the outpatient clinic. Asthmatic participants further filled in the Asthma Control Questionnaire (Juniper et al., 1999) during the visit.

# S2 Data preprocessing

The following section serves as the extended version of the data preprocessing section in the main manuscript.

The mass spectral data were preprocessed almost identically as previously described by our group (Weber et al., 2020). We repeat the procedure here for the sake of completeness. The proprietary .wiff data files containing mass spectra from the measurements were converted to the open .mzXML file format using MSConvert (ProteoWizard v3.0.2) (Kessner et al., 2008) and further processed in R v4.1.1 (R Foundation for Statistical Computing, Vienna, Austria). All mass spectra were resampled using piecewise cubic Hermite interpolation (Fritsch and Carlson, 1980) onto a linearly spaced *m/z*-axis with a resolution of 0.0005 (*m/z* range 50-500, 9 x 10^8^ data points). Total ion chromatograms (TIC) were calculated by trapezoidal integration and used to differentiate between mass spectra in exhaled breath and pauses between the exhalations.

For each subject, peak picking was performed on the mass spectrum averaged over the scans generated during exhalations where the minimum average intensity for a peak to be selected was set to 100 counts per second (cps) in both the negative and the positive ionization mode. In order to compensate for small variations in the peak positions across the experiments all recorded *m/z* features were combined into one single list from which a kernel density estimate (Gaussian kernel, bandwidth = 0.0025) was computed. Local maxima of the smoothed density function were used to define the final *m/z* feature list representative for all samples. Centroiding of the final peak list was done by trapezoidal integration (mass window *m/z* ± ∆*m/z*, ∆*m/z* = 0.0025) yielding in time traces of intensities of the *m/z* features for each measurement. To exclude *m/z* features which are not associated with exhalations, a simple linear regression model was built for each measured sample between the standardized TIC and the standardized *m/z* feature’s timetrace. The features with estimated slope < 0 were excluded from the list. Additionally, the ratio of the sum of squares of the predicted values by simple linear regression model of the *m/z* feature over the scans and the sum of squares of the standardized TIC was used as a filtering parameter to further reduce the number features to the ones associated with the exhalation maneuver. The minimum threshold for the ratio was set to 0.5 in both ionization modes. Next, to exclude the features which were inconsistently measured over all samples, the *m/z* features which passed the filters above in at least 30% of all the samples were kept for further processing. Finally, the intensities of the remaining *m/z* features in each measurement were averaged over scans corresponding to exhalations, normalized to the TIC, log_2_ transformed and arranged into an n x k matrix of breath profiles with n the number of subjects and k the number of *m/z* features.

# S3 Statistical analysis

The following section serves as the extended version of the statistical analysis section in the main manuscript.

Due to a lengthy study conducted in a laboratory where also other experiments were being executed, it was assumed that unknown technical confounders had impacted the intensity levels of the *m/z* features. In order to adjust for such unmodeled factors, surrogate variable analysis algorithm (SVA) by Leek and Storey (Leek and Storey, 2007, 2008) was applied to the data matrix of the breath profiles (R package “sva” version 3.40.0). The number of surrogate variables (SVs) was estimated automatically using the permutation procedure (Buja and Eyuboglu, 1992) (corresponding to method “BE” in the function “sva” of the same R package). For statistical inference the SVs were included as covariates into the linear models of individual *m/z* features, *i.e.*


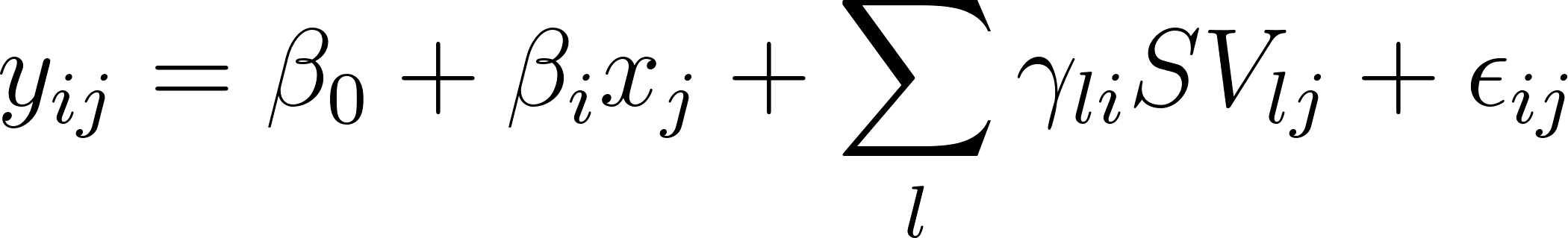


(see in (Fritsch and Carlson, 1980; Leek et al., 2012)) where y_ij_ represents the intensity of the i^th^ feature in the j^th^ sample and x_j_ the dichotomous outcome (asthmatic or healthy) of the j^th^ sample. The identification of statistically significant features comparing asthmatic and non-asthmatic samples while controlling for the SVs was conducted using the empirical Bayes moderated t-statistics test (Smyth, 2004). The calculation of the *p*-values and log-fold changes were performed using the “limma” package (v3.52.0) with function “lmFit”, “eBayes”, and “topTable” in R (Ritchie et al., 2015; Phipson et al., 2016). Note that, according to the implementation in "limma" the log-fold-change for each m/z feature i is defined as the estimated regression coefficients βi in the fitted linear model above. To control for the false discovery rate (FDR) Benjamini-Hochberg procedure (Benjamini and Hochberg, 1995) was applied to convert p-values into adjusted p-values with the significance threshold set to the adjusted p-value of 0.05. Principal component analysis was conducted on the subset of *m/z* features with the scores of the first two principal components plotted for a low dimensional presentation of the samples. For visualization purposes (PCA, heatmaps and boxplots) the SVs were regressed out from the data (Jaffe et al., 2015).

The predictive ability of the exhaled breath profiles to classify the samples as asthmatic or non-asthmatic was assessed in a 10 times repeated 10-fold cross-validation. In each iteration of the cross-validation support vector machine algorithm (SVM) (Cortes and Vapnik, 1995) with linear kernel and the soft margin constant C = 1 was trained to predict the unlabeled left-out sample where batch correction wasconducted with frozen surrogate variable analysis (fSVA) (Parker et al., 2014). All preprocessing steps needed to derive the breath profiles were repeated in each cross-validation iteration on the training data sets to avoid information leakage from the testing data set (Varma and Simon, 2006). Despite the insensitivity of the SVM algorithm to high dimensional data we trained the algorithm in each iteration of the cross-validation on a smaller set of selected features, where Boruta scheme (Kursa et al., 2010) for feature selection was applied (R package “Boruta” version 7.0.0). The settings for the *Boruta* function were set to the default values as provided by the package and *TentativeRoughFix* function from the same package was used to additionally either confirm or reject the features which were tagged as tentative by the *Boruta* function. The predictions on the left-out sample in the cross-validation were assigned probabilities scores (Lin et al., 2007) and used to populate the receiver operating characteristic (ROC) space by varying the cut-off threshold. The resulting ROC curves for each cross-validation loop (Fawcett, 2006) were then used to calculate the average ROC curve over all cross-validation loops by applying vertical averaging (Fawcett, 2006). The overall performance of the classification was then reported by the average area under the curve (AUC) over all cross-validation loops.

# S4 Compound identification

For putative compound identification, MS^2^ spectra of a large amount of the significant *m/z* features were recorded directly from exhaled breath using the same instrument set-up as for participant measurements. The MS^2^ spectra were converted to .mgf file format by MSConvert (ProteoWizard 3.0.2) (Kessner et al. 2008) and analyzed by applying an adapted version of the methodology for putative compound identification as described by Kaeslin *et al.* (Kaeslin et al., 2021). The multi-level process assigned the most likely molecular formulae and chemical structures to the respective *m/z* feature by using the open source software SIRIUS (v4.9.9) (Dührkop et al., 2019). The final chemical structures were assigned with an identification level (1 to 4) that reflects the certainty of identification according to Schymanski (Schymanski et al., 2014). The workflow was adapted to this specific dataset and is represented in Figure S1.

Detailed description for compound identification of significant *m/z* features elevated in the allergic asthmatic and healthy control group, respectively:

During the entire identification process, features significant for each study cohort were analyzed separately.

1. Isotopes and SESI characteristics were identified based on the calculated differences between *m/z* features. The resulting relationships were indicated in Table E2.
2. The selected MS^2^ spectra were analyzed by SIRIUS with settings as specified (Figure E1).
3. The suggested molecular formulae were ranked by their SIRIUS score. First, the formula with the highest SIRIUS score was considered. The final molecular formula and ionization species were determined based on the proposed chemical structures and their plausibility.
4. Assignment of the putative chemical structures based on the CSI:FingerID (Kai et al., 2015) score ranking from SIRIUS and the biological relevance:
   1. Resulting compound suggestions were excluded based on the specified exclusion criteria (Figure E1, Table E1).
   2. All suggested structures were ranked according to their CSI:FingerID score.
   3. First iteration: The chemical structure with the highest CSI:FingerID score was selected for each MS^2^ spectrum.
   4. Compounds were grouped into biochemical pathways of the KEGG database (Kanehisa and Goto, 2000) or into chemical families.
   5. Second iteration: If the highest ranked chemical structure was not part of an enhanced pathway or chemical family, all structures within a small range of the highest CSI:FinderID score were considered. If an alternative suggestion corresponded to a pathway or chemical family found in 4.3), it was selected instead and the highest ranked structure neglected.
   6. Pathways or chemical families represented by at least three entries were considered important during the compound identification process and their chemical structures were finally selected.
   7. Column S of Table S2 indicates the rank of the chemical structure that was selected out of the total amount of possible and allowed hit suggestions.
   8. For all remaining features resulting in compounds of a non-important labelled pathway/ family, the best ranked chemical structure was chosen (Table E2).
5. *m/z* features without chemical structure hits in SIRIUS were subjected to functional analysis by MetaboAnalyst applying the mummichog algorithm for pathway enrichment analysis (Li et al., 2013; Pang et al., 2021) (together with all significant *m/z* features of the respective cohort, settings as specified in Figure E1); only pathway/compound families with at least three entries were considered.
   1. If this resulted in compound suggestions from a pathway/ chemical family which was already represented before, these compounds were selected as additional putative candidates (ID level 4).
   2. If this resulted in several compound suggestions from the same pathway (that was already represented before), all suggestions were listed.
6. The detected *m/z* features were also matched to previously identified compounds in the literature. Thus, some features which have been excluded previously based on the exclusion criteria (Table E1) were eventually included in the identified list (Table E2) by literature match.

# S5 Supplementary Figures


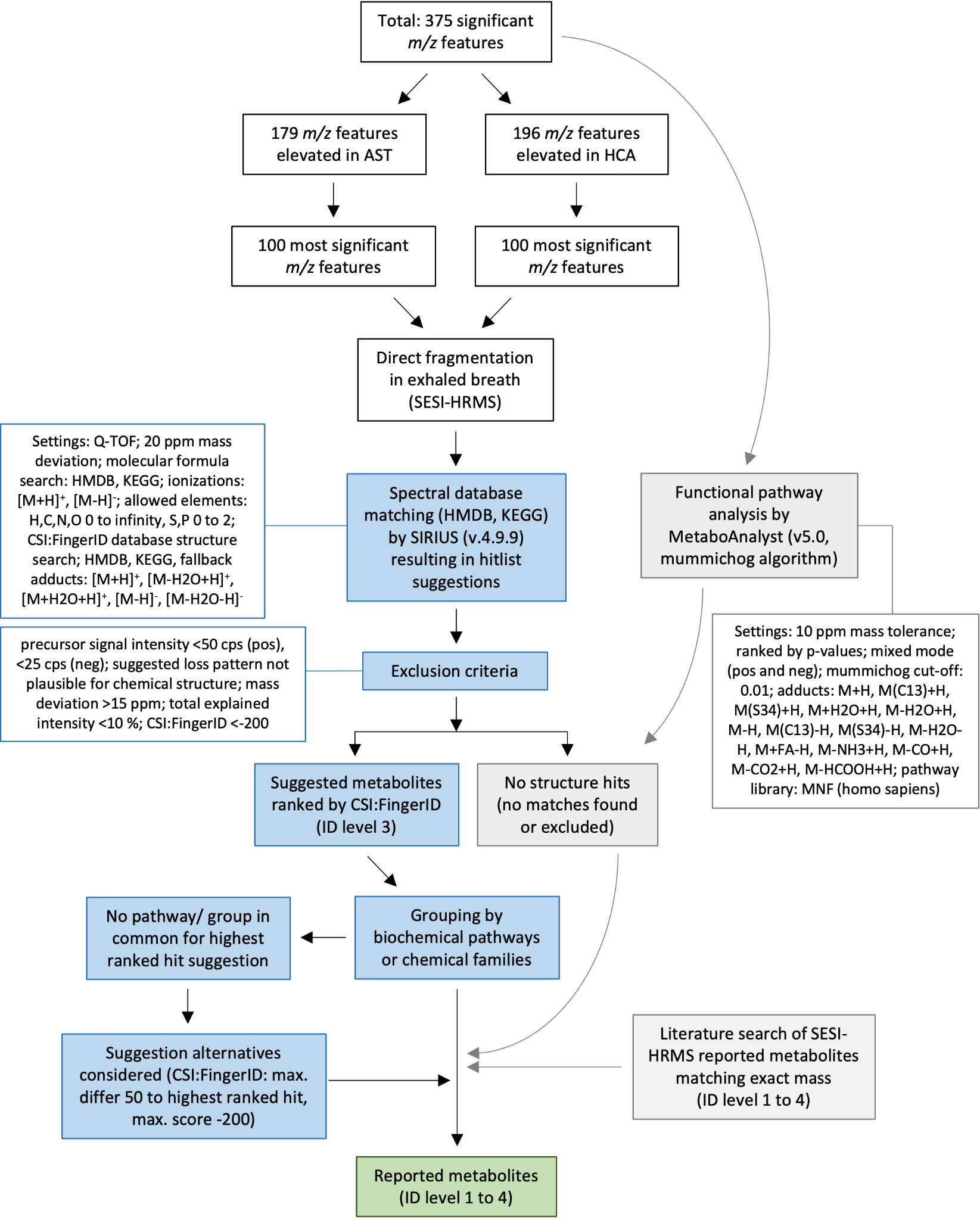


**Supplementary Figure 1.** Scheme of the applied approach for compound identification, Metabolites were putatively identified by fragment spectra matching (SIRIUS) with the HMDB and KEGG database (MS^2^, filled blue boxes), applied settings: framed blue boxes. The resulting compound suggestions were ranked by their CSI-FingerID score and the highest ranked suggestions grouped by their relation to biochemical pathways or chemical families. Within a range of +50 of the CSI:FingerID score, also lower ranked suggestions were considered. Grey boxes: Identification by literature match or by additional functional pathway analysis based on exact mass (MS^1^) using MetaboAnalyst, performed on *m/z* features which didn’t result in plausible or allowed compound suggestions during spectral database matching.


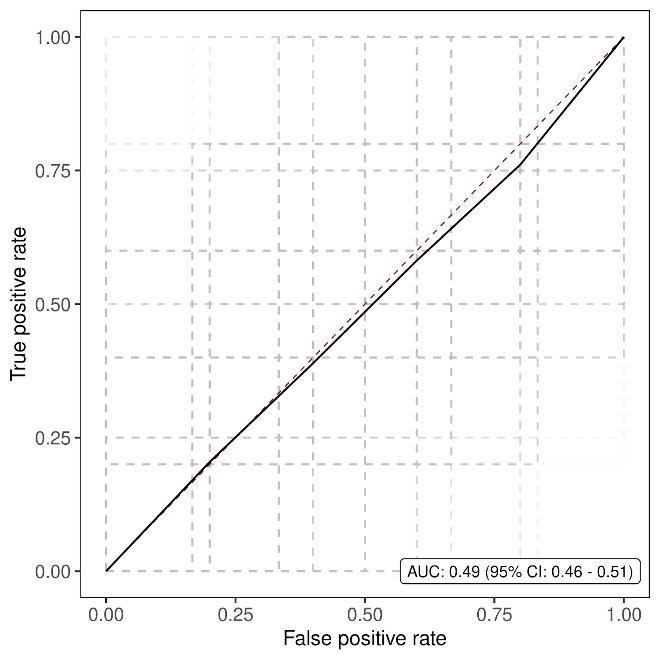


**Supplementary Figure 2.** Permutation test for the classification algorithm. Receiver operating characteristic curve (ROC) of our classification pipeline, repeated 1000 times while permuting the labels randomly in cross-validation. The average ROC (bold black line) curve shows an AUC of approximately 0.49, showing that the classification algorithm is unbiased. Bold black line: average ROC curve; red dashed line: line of no discrimination; dotted gray lines (transparency set to 90% for better visual representation): every single ROC curve from each single training and testing data split arising during permutation test.


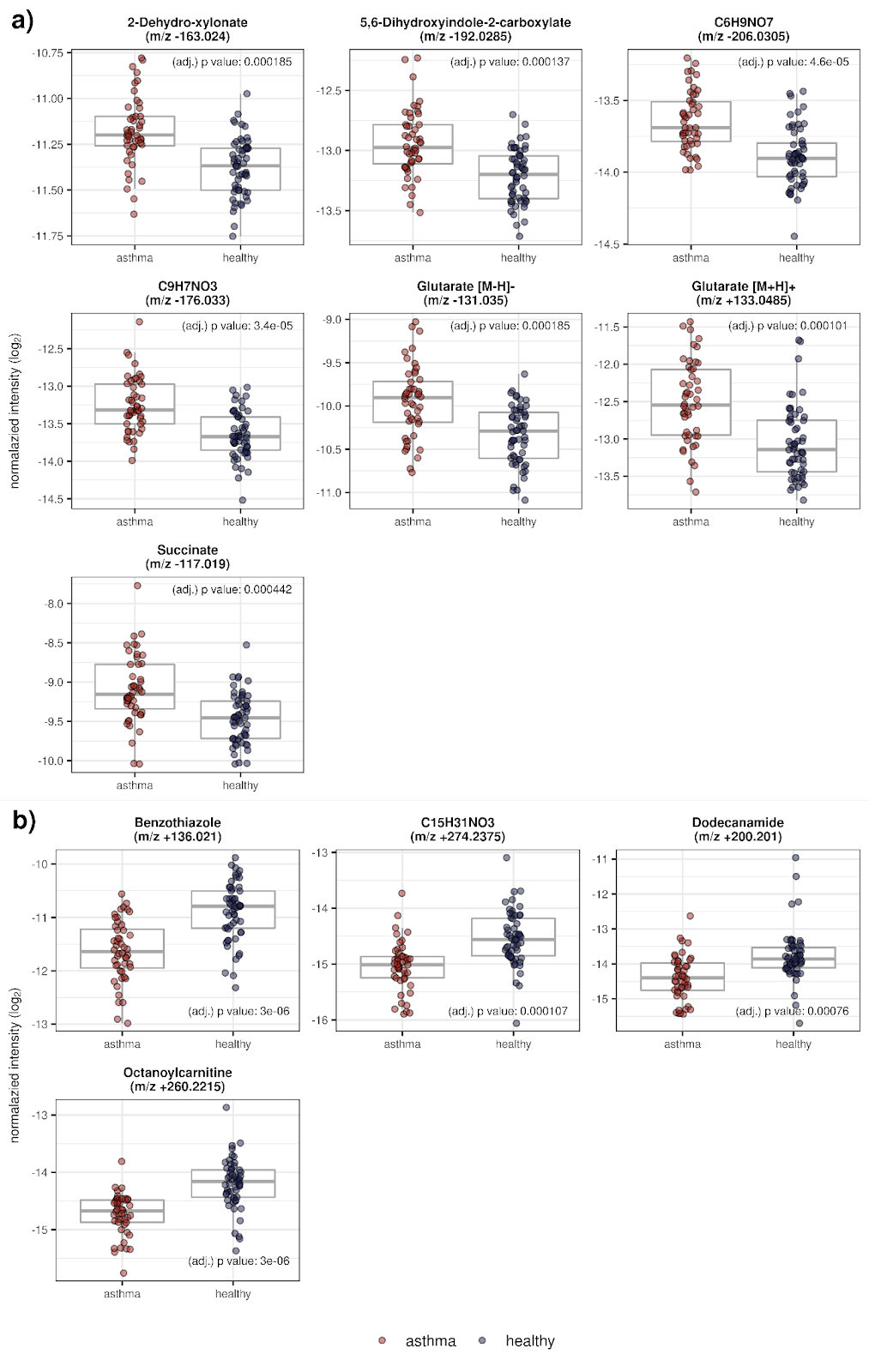


**Supplementary Figure 3.** Boxplots of the most frequently selected compounds as predictors during cross-validation. **a)** Compounds which were upregulated in the asthmatic patients. **b)** compounds which were upregulated in the healthy controls.


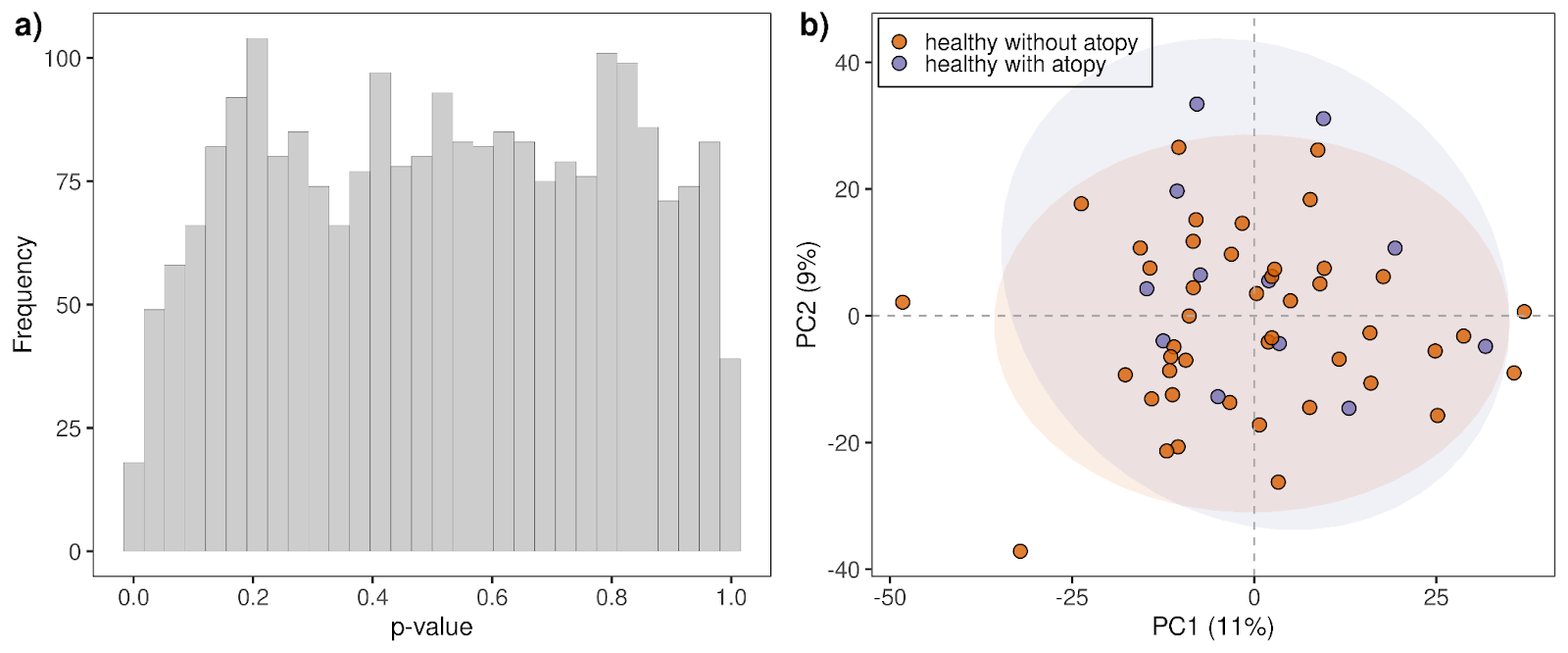


**Supplementary Figure 4.** Subgroup analysis of 12 healthy controls with atopy versus 44 healthy controls without atopy. The distribution of p-values and subsequent correction for FDR showed no significant difference between healthy probands with and without atopy. In addition, PCA plots showed no clear distinction between the two groups. Notably, the subgroups were quite unbalanced (12 vs 44) which was not ideal for an analysis. **a)** Distribution of p-values for the subgroups of healthy controls when assessing atopy vs. no atopy. **b)** PCA plot consisting of the scores of the first two principal components. Violet dots: healthy controls with atopy. Orange dots: healthy controls without atopy.

# Supplementary Tables

**Supplementary Table 1.** Exclusion criteria of MS2 spectra applied during compound identification.

| **Exclusion criteria** | **Excluded in asthmatics (#)** | **Excluded in healthy controls (#)** |
| --- | --- | --- |
| Acquisition error | 3 | 1 |
| Low precursor signal* | 16 | 7 |
| Mass error > 15 ppm | 4 | 4 |
| Identification score CSI < -200 | 4 | 11 |
| Fragment intensity match < 10% | 4 | 13 |
| Compound class: drugs | 5 | 5 |
| No metabolite suggestion | 12 | 31 |

* Cut off: precursor signal ≤ 50 cps in positive ion mode, ≤ 25 cps in negative ion mode.

**Supplementary Table 2.** Full table for compound identification on all 375 discriminative m/z features.

The table is attached as a separate excel file due to its size.

**Supplementary Table 3.** Accuracy estimation from 10 times repeated 10-fold cross-validation. Table summarizing AUC, accuracy, specificity and sensitivity resulting from the 10 times repeated 10-fold cross-validation. 95% confidence intervals are included (calculated using bootstrapping with 10000 repetitions).

| Average AUC | 0.83 (95% CI: 0.73 - 0.92) |
| --- | --- |
| Average accuracy | 78.0% (95% CI: 69.7% - 86.0%) |
| Average sensitivity | 74.0% (95% CI: 60.6% - 867%) |
| Average specificity | 81.6% (95% CI: 71.0% - 91.4%) |

**Supplementary Table 4.** All selected features as predictors in the cross-validation (10 repetitions of 10-fold cross-validation).

| ***m/z*** | **Charge** | ***p*-value**  **(adj.)** | **Upreg.**  **in** | **Compound** | **Pathway /**  **chemical family** | **Selection**  **frequency** | **Final**  **Model** |
| --- | --- | --- | --- | --- | --- | --- | --- |
| 163.024 | neg | 0.00018 | AST | 2-Dehydro-xylonate | Monosaccharides and metabolites | 100/100 | 1 |
| 136.021 | pos | 0.00000 | HCA | Benzothiazole | Heterocyclic compounds | 100/100 | 1 |
| 260.2215 | pos | 0.00000 | HCA | Octanoylcarnitine | Fatty acyl carnitines | 100/100 | 1 |
| 176.033 | neg | 0.00003 | AST | C9H7NO3 |  | 99/100 | 1 |
| 206.0305 | neg | 0.00005 | AST | C6H9NO7 |  | 99/100 | 1 |
| 133.0485 | pos | 0.00010 | AST | Glutarate [M+H]+ | Lysine degradation | 96/100 | 1 |
| 117.019 | neg | 0.00044 | AST | Succinate | Lysine degradation | 95/100 | 1 |
| 200.201 | pos | 0.00076 | HCA | Dodecanamide | Fatty amides | 93/100 | 1 |
| 274.2375 | pos | 0.00011 | HCA | C15H31NO3 |  | 92/100 | 1 |
| 192.0285 | neg | 0.00014 | AST | 5,6-Dihydroxyindole-2-carboxylate | Tyrosine metabolism | 91/100 | 1 |
| 131.035 | neg | 0.00018 | AST | Glutarate [M-H]- | Lysine degradation | 90/100 | 1 |
| 181.086 | neg | 0.00005 | AST | ω-Oxodecadienoic acid | Fatty acid metabolites | 88/100 | 1 |
| 91.0035 | neg | 0.00014 | HCA | Formic acid dimer |  | 87/100 | 1 |
| 288.253 | pos | 0.00028 | HCA | Lauroyl diethanolamide | Fatty amides | 87/100 | 1 |
| 149.0605 | neg | 0.00006 | AST | 2-Phenylpropionate | Phenylpropanoic acid | 86/100 | 1 |
| 160.133 | pos | 0.00202 | HCA | 4-Hydroxy-2-octenal | Aldehydes | 86/100 | 1 |
| 153.0555 | neg | 0.00034 | AST | 5-Ethylbenzene-1,2,3-triol | Aromatic compound | 85/100 | 1 |
| 197.081 | neg | 0.00042 | AST | 2,7-Dimethyl-2,4-octadienedioic acid | Fatty acid metabolites | 84/100 | 1 |
| 92.0065 | neg | 0.00018 | HCA | Formic acid dimer (13C isotope) |  | 82/100 | 1 |
| 179.0185 | neg | 0.00092 | AST | 2,5-Dimethyl-1,4-dithiane-2,5-diol | Dithiane | 81/100 | 1 |
| 132.1015 | pos | 0.00047 | HCA | 4-Hydroxy-2-hexenal | Aldehydes | 81/100 | 0 |
| 154.0255 | neg | 0.00034 | AST | N-Methylethanolaminium phosphate | Glycerophospholipid metabolism | 74/100 | 0 |
| 153.019 | neg | 0.00266 | AST | 2,6-Dihydroxybenzoate | Benzoate degradation | 73/100 | 0 |
| 155.0315 | pos | 0.00004 | AST |  |  | 72/100 | 1 |
| 180.014 | neg | 0.00087 | AST |  |  | 68/100 | 0 |
| 272.2235 | pos | 0.00109 | HCA |  |  | 68/100 | 0 |
| 165.0545 | neg | 0.00127 | AST | cis-3-(1-Carboxy-ethyl)-3,5-cyclo-hexadiene-1,2-diol |  | 63/100 | 0 |
| 232.1905 | pos | 0.00409 | HCA |  |  | 62/100 | 0 |
| 271.2265 | pos | 0.00065 | HCA | 10,16-Dihydroxyhexadecanoic acid | Fatty acids | 61/100 | 0 |
| 169.05 | neg | 0.00064 | AST | 2-Oxosuberate (2-oxooctanedioic acid) | 2-Oxocarboxylic acid metabolism | 60/100 | 1 |
| 129.019 | neg | 0.00076 | AST | 2-Hydroxyglutarate (2-hydroxypentanedioc acid) | Lysine degradation | 55/100 | 0 |
| 171.0295 | neg | 0.00427 | AST | 3-Dehydroquinate | Phenylalanine, tyrosine and tryptophan biosynthesis | 54/100 | 0 |
| 188.0335 | neg | 0.00093 | AST | Quisqualic acid |  | 54/100 | 0 |
| 134.058 | neg | 0.03126 | AST |  |  | 52/100 | 1 |
| 162.0755 | pos | 0.00697 | AST | 2-Aminoadipate (2-aminohexanedioic acid) | Lysine degradation, 2-oxocarboxylic acid metabolism | 52/100 | 0 |
| 158.0535 | neg | 0.00443 | AST | Heptenedioic acid | Fatty acid metabolites | 51/100 | 0 |
| 96.9595 | neg | 0.00188 | AST | Sulfate | Sulfur metabolism, purine metabolism | 50/100 | 1 |
| 136.133 | pos | 0.00043 | HCA |  |  | 50/100 | 0 |
| 248.1125 | pos | 0.00043 | HCA | Linamarin | Glycosides | 49/100 | 1 |
| 260.185 | pos | 0.01953 | HCA |  |  | 49/100 | 0 |
| 276.2165 | pos | 0.03083 | HCA |  |  | 46/100 | 1 |
| 130.024 | neg | 0.00149 | AST | (3-Nitroamino)alanine | Alpha amino acid | 45/100 | 1 |
| 167.0685 | pos | 0.00093 | AST | 3-Ethoxybenzoate | Aromatic compound | 45/100 | 1 |
| 195.981 | pos | 0.00076 | HCA |  |  | 42/100 | 1 |
| 222.0255 | neg | 0.00634 | AST |  |  | 41/100 | 1 |
| 95.9515 | neg | 0.00093 | AST |  |  | 41/100 | 0 |
| 93.0075 | neg | 0.00276 | HCA | Formic acid dimer (18O isotope) |  | 40/100 | 1 |
| 114.091 | pos | 0.00266 | HCA |  |  | 40/100 | 0 |
| 194.031 | neg | 0.00266 | AST |  |  | 39/100 | 0 |
| 220.1905 | pos | 0.00414 | HCA | 11-Aminoundecanoic acid | Fatty acids | 38/100 | 1 |
| 170.096 | pos | 0.00202 | HCA | Diphenylamine | Benzene | 37/100 | 0 |
| 129.033 | pos | 0.00028 | AST |  |  | 36/100 | 0 |
| 111.947 | neg | 0.00208 | AST |  |  | 35/100 | 0 |
| 183.0295 | neg | 0.00381 | AST | Succinylacetoacetate | Tyrosine metabolism | 34/100 | 0 |
| 190.045 | neg | 0.00702 | AST |  |  | 33/100 | 0 |
| 211.0605 | neg | 0.00129 | AST |  |  | 32/100 | 0 |
| 110.0805 | pos | 0.00298 | HCA |  |  | 32/100 | 0 |
| 137.009 | neg | 0.00964 | HCA | 5-Diazouracil | Uracil derivative | 30/100 | 1 |
| 195.0655 | neg | 0.00160 | AST |  |  | 30/100 | 1 |
| 104.07 | pos | 0.00018 | HCA | 4-Aminobutanoate | Arginine and proline metabolism | 30/100 | 0 |
| 96.9925 | neg | 0.01129 | HCA | Fumarate;  Maleate | Arginine biosynthesis; Carboxylic acids | 29/100 | 0 |
| 260.1485 | pos | 0.00210 | HCA |  |  | 29/100 | 0 |
| 152.035 | neg | 0.00409 | AST | 4-Methyl-2-nitrophenol | Nitroaromatic compound | 28/100 | 0 |
| 151.0585 | pos | 0.00127 | AST | Arabinose | Monosaccharide, ascorbate and aldarate metabolism, pentose and glucuronate interconversions, amino sugar and nucleotide sugar metabolism | 28/100 | 0 |
| 61.039 | pos | 0.00757 | HCA | Urea | Arginine and proline metabolism | 28/100 | 0 |
| 148.0395 | neg | 0.00414 | AST |  |  | 27/100 | 0 |
| 90.721 | neg | 0.00307 | HCA |  |  | 27/100 | 1 |
| 316.2845 | pos | 0.00008 | HCA | Palmitoleoylethanolamde (POEA) | Fatty amides | 27/100 | 1 |
| 136.0395 | neg | 0.00315 | AST |  |  | 26/100 | 0 |
| 258.098 | pos | 0.00971 | HCA | Cardiospermin | Glycosides | 26/100 | 0 |
| 204.062 | neg | 0.00260 | AST |  |  | 24/100 | 0 |
| 220.046 | neg | 0.00260 | AST | Histidinol phosphate | Histidine metabolism | 24/100 | 0 |
| 222.17 | pos | 0.00715 | HCA |  |  | 24/100 | 0 |
| 60.0805 | pos | 0.00825 | HCA | 4-Aminobutanal;  4-Aminobutanoate | Arginine and proline metabolism | 24/100 | 0 |
| 222.097 | pos | 0.01135 | HCA | Aldehydo-N-acetyl-D-glucosamine | Glycosides | 23/100 | 0 |
| 149.0245 | neg | 0.01633 | AST | 3,4-Dihydroxymandelaldehyde | Tyrosine metabolism | 22/100 | 0 |
| 135.0235 | pos | 0.00278 | AST |  |  | 20/100 | 0 |
| 163.0495 | neg | 0.01135 | AST |  |  | 19/100 | 0 |
| 236.1125 | pos | 0.00756 | HCA |  |  | 19/100 | 0 |
| 100.9875 | neg | 0.01846 | HCA |  |  | 18/100 | 0 |
| 218.0305 | neg | 0.00940 | AST | 4-Amino-2-methyl-5-(phosphooxymethyl)pyrimidine | Thiamine metabolism | 18/100 | 0 |
| 191.1635 | pos | 0.02516 | HCA |  |  | 18/100 | 0 |
| 166.0485 | neg | 0.00505 | AST | Nitrophenol derivative | Nitroaromatic compound | 17/100 | 0 |
| 178.0355 | neg | 0.01289 | AST | 2-Amino-3,4-dihydroxypentanedioic acid | Fatty acid metabolites | 17/100 | 0 |
| 232.046 | neg | 0.00410 | AST |  |  | 17/100 | 0 |
| 147.0645 | pos | 0.00702 | AST | Adipic acid (hexanedioic acid) | Fatty acid metabolites | 17/100 | 0 |
| 132.043 | neg | 0.00305 | AST | Ureidoglycine | Purine metabolism | 16/100 | 0 |
| 94.9805 | neg | 0.00443 | AST | Methanesulfonic acid | Sulfur metabolism | 16/100 | 0 |
| 108.08 | pos | 0.00188 | HCA | ortho-Toluidine | Benzene | 16/100 | 0 |
| 156.1015 | pos | 0.00075 | HCA | 4-(aminoethyl)phenol | Aromatics | 16/100 | 0 |
| 177.075 | pos | 0.00171 | AST | Succinylacetone | Tyrosine metabolism | 16/100 | 0 |
| 208.1175 | pos | 0.01690 | HCA |  |  | 16/100 | 0 |
| 210.133 | pos | 0.00702 | HCA |  |  | 16/100 | 0 |
| 170.02 | neg | 0.01229 | AST |  |  | 15/100 | 0 |
| 193.035 | neg | 0.01963 | AST | Glucuronate | Monosaccharides and metabolites | 15/100 | 0 |
| 216.051 | neg | 0.01229 | AST |  |  | 15/100 | 0 |
| 251.0065 | neg | 0.00364 | AST |  |  | 15/100 | 0 |
| 178.1435 | pos | 0.01733 | HCA |  |  | 15/100 | 0 |
| 186.0185 | pos | 0.01785 | HCA |  |  | 15/100 | 0 |
| 180.1225 | pos | 0.00595 | HCA |  |  | 14/100 | 0 |
| 122.9805 | neg | 0.02933 | AST |  |  | 13/100 | 0 |
| 123.045 | neg | 0.02644 | AST |  |  | 13/100 | 0 |
| 125.06 | neg | 0.01953 | AST | 7-Oxoheptanoic acid | Fatty acid metabolites | 13/100 | 0 |
| 140.035 | neg | 0.01321 | AST | 2-Aminomuconate semialdehyde | Tryptophan metabolism, aminobenzoate degradation | 13/100 | 0 |
| 146.024 | neg | 0.01525 | AST |  |  | 13/100 | 0 |
| 164.035 | neg | 0.03260 | AST |  |  | 13/100 | 0 |
| 227.0415 | neg | 0.03443 | AST |  |  | 13/100 | 0 |
| 172.133 | pos | 0.00039 | HCA | 4-Hydroxy-2,6-nonadienal | Aldehydes | 13/100 | 0 |
| 199.058 | pos | 0.00103 | AST | cis-(Homo)3-aconitate | 2-Oxocarboxylic acid metabolism | 13/100 | 1 |
| 234.025 | neg | 0.01547 | AST |  |  | 12/100 | 0 |
| 145.049 | pos | 0.00161 | AST | Galactose | Monosaccharides and metabolites | 12/100 | 0 |
| 195.1225 | pos | 0.01586 | HCA |  |  | 12/100 | 0 |
| 140.9825 | neg | 0.04404 | HCA |  |  | 11/100 | 0 |
| 146.0545 | neg | 0.04044 | AST | 2-Aceto-2-hydroxybutanoate | 2-Oxocarboxylic acid metabolism | 11/100 | 0 |
| 197.0305 | neg | 0.03152 | AST |  |  | 11/100 | 0 |
| 173.0405 | pos | 0.00149 | AST |  |  | 11/100 | 0 |
| 175.06 | pos | 0.00697 | AST |  |  | 11/100 | 0 |
| 167.071 | neg | 0.02644 | AST | ω-Oxononadienoic acid | Fatty acid metabolites | 10/100 | 0 |
| 332.22 | neg | 0.00186 | HCA |  |  | 10/100 | 0 |
| 228.159 | pos | 0.00519 | HCA |  |  | 10/100 | 0 |
| 71.068 | pos | 0.00260 | HCA | Pyrroline | Heterocyclic compounds | 10/100 | 0 |
| 315.218 | neg | 0.00904 | HCA |  |  | 9/100 | 0 |
| 130.0855 | pos | 0.00492 | HCA |  |  | 9/100 | 0 |
| 146.117 | pos | 0.02103 | HCA | 4-Hydroxy-2-heptenal | Aldehydes | 9/100 | 0 |
| 186.1845 | pos | 0.00756 | HCA | beta-Skytanthine | Alkaloids | 9/100 | 0 |
| 110.0245 | neg | 0.03789 | AST |  |  | 8/100 | 0 |
| 111.0175 | neg | 0.04353 | AST |  |  | 8/100 | 0 |
| 87.019 | neg | 0.02989 | AST |  |  | 8/100 | 0 |
| 189.4175 | neg | 0.02921 | AST |  |  | 7/100 | 0 |
| 248.041 | neg | 0.01580 | AST |  |  | 7/100 | 0 |
| 260.041 | neg | 0.04446 | AST |  |  | 7/100 | 0 |
| 333.2255 | neg | 0.00202 | HCA |  |  | 7/100 | 0 |
| 115.0945 | pos | 0.00599 | HCA |  |  | 7/100 | 0 |
| 246.206 | pos | 0.02304 | HCA |  |  | 7/100 | 0 |
| 141.029 | neg | 0.02304 | AST |  |  | 6/100 | 0 |
| 235.118 | neg | 0.01075 | AST |  |  | 6/100 | 0 |
| 150.0755 | pos | 0.04327 | AST |  |  | 6/100 | 0 |
| 208.1905 | pos | 0.02319 | HCA |  |  | 6/100 | 0 |
| 228.149 | pos | 0.02464 | HCA |  |  | 6/100 | 0 |
| 236.075 | pos | 0.04530 | HCA |  |  | 6/100 | 0 |
| 246.133 | pos | 0.03865 | HCA |  |  | 6/100 | 0 |
| 256.1915 | pos | 0.04626 | HCA |  |  | 6/100 | 0 |
| 129.0295 | neg | 0.01239 | AST | beta-L-Aspartylhydroxamate | Alpha amino acid | 5/100 | 0 |
| 143.0345 | neg | 0.03634 | AST |  |  | 5/100 | 0 |
| 186.0405 | neg | 0.01054 | AST |  |  | 5/100 | 0 |
| 68.998 | neg | 0.02638 | HCA |  |  | 5/100 | 0 |
| 148.06 | pos | 0.00770 | AST | Glutamate | 2-Oxocarboxylic acid metabolism | 5/100 | 0 |
| 168.1225 | pos | 0.00702 | HCA |  |  | 5/100 | 0 |
| 229.001 | pos | 0.03171 | HCA |  |  | 5/100 | 0 |
| 254.211 | pos | 0.01510 | HCA |  |  | 5/100 | 0 |
| 147.0295 | neg | 0.02522 | AST |  |  | 4/100 | 0 |
| 155.071 | neg | 0.02682 | AST | ω-Oxooctenoic acid | Fatty acid metabolites | 4/100 | 0 |
| 202.036 | neg | 0.01424 | AST |  |  | 4/100 | 0 |
| 172.0595 | pos | 0.02440 | AST |  |  | 4/100 | 0 |
| 199.9125 | pos | 0.03590 | HCA |  |  | 4/100 | 0 |
| 228.196 | pos | 0.02379 | HCA | 4-Hydroxy-2,6-tridecadienal | Aldehydes | 4/100 | 0 |
| 70.0645 | pos | 0.00288 | HCA | Pyrroline | Heterocyclic compounds | 4/100 | 0 |
| 209.03 | neg | 0.02438 | AST | Glucarate | Monosaccharides and metabolites | 3/100 | 0 |
| 212.9995 | neg | 0.02812 | HCA |  |  | 3/100 | 0 |
| 215.0205 | neg | 0.04535 | AST |  |  | 3/100 | 0 |
| 317.2335 | neg | 0.00625 | HCA |  |  | 3/100 | 0 |
| 133.028 | pos | 0.03083 | AST |  |  | 3/100 | 0 |
| 144.1375 | pos | 0.00154 | HCA | gamma-Coniceine | Alkaloids | 3/100 | 0 |
| 152.1275 | pos | 0.00308 | HCA |  |  | 3/100 | 0 |
| 159.0645 | pos | 0.00150 | AST | 3-Isopropylmalate | 2-Oxocarboxylic acid metabolism | 3/100 | 0 |
| 162.1485 | pos | 0.02812 | HCA |  |  | 3/100 | 0 |
| 185.0595 | pos | 0.00702 | AST |  |  | 3/100 | 0 |
| 193.13 | pos | 0.02510 | HCA | L-Arginine | Arginine and proline metabolism | 3/100 | 0 |
| 274.2015 | pos | 0.01586 | HCA |  |  | 3/100 | 0 |
| 119.071 | neg | 0.01988 | AST | Pentanoic acid | Fatty acid metabolites | 2/100 | 0 |
| 120.0445 | neg | 0.02861 | AST |  |  | 2/100 | 0 |
| 160.04 | neg | 0.02881 | AST |  |  | 2/100 | 0 |
| 183.049 | neg | 0.04284 | AST |  |  | 2/100 | 0 |
| 196.0455 | neg | 0.04949 | AST |  |  | 2/100 | 0 |
| 230.0305 | neg | 0.01586 | AST |  |  | 2/100 | 0 |
| 63.962 | neg | 0.01316 | AST |  |  | 2/100 | 0 |
| 97.029 | neg | 0.01368 | AST | Glutarate semialdehyde (ω-oxopentanoic acid) | Lysine degradation | 2/100 | 0 |
| 105.0365 | pos | 0.00641 | AST | Divinyl sulfide | Thioenol ether | 2/100 | 0 |
| 222.1335 | pos | 0.01552 | HCA |  |  | 2/100 | 0 |
| 234.1335 | pos | 0.01439 | HCA |  |  | 2/100 | 0 |
| 235.9345 | pos | 0.04578 | HCA |  |  | 2/100 | 0 |
| 256.154 | pos | 0.01029 | HCA |  |  | 2/100 | 0 |
| 98.0595 | pos | 0.00266 | HCA | Proline | Arginine and proline metabolism | 2/100 | 0 |
| 157.0505 | neg | 0.02304 | AST | Heptenedioic acid | Fatty acid metabolites | 1/100 | 0 |
| 158.02 | neg | 0.03439 | AST |  |  | 1/100 | 0 |
| 161.082 | neg | 0.02410 | AST |  |  | 1/100 | 0 |
| 163.039 | neg | 0.01342 | AST | 4-Coumarate | Tyrosine metabolism | 1/100 | 0 |
| 179.036 | neg | 0.02136 | AST | 4-Hydroxyphenylpyruvate;  4-Hydroxy-enol-phenylpyruvate;  3-Methoxy-4-hydroxymandelate | Tyrosine metabolism | 1/100 | 0 |
| 181.0505 | neg | 0.01225 | AST | 4-Hydroxyphenyllactate | Tyrosine metabolism | 1/100 | 0 |
| 199.025 | neg | 0.02251 | AST | 4-Maleylacetoacetate;  4-Fumarylacetoacetate | Tyrosine metabolism | 1/100 | 0 |
| 222.062 | neg | 0.02251 | AST |  |  | 1/100 | 0 |
| 226.0345 | neg | 0.03342 | AST |  |  | 1/100 | 0 |
| 252.0315 | neg | 0.01785 | AST |  |  | 1/100 | 0 |
| 252.048 | neg | 0.03619 | AST |  |  | 1/100 | 0 |
| 271.2275 | neg | 0.01229 | HCA |  |  | 1/100 | 0 |
| 331.212 | neg | 0.02136 | HCA |  |  | 1/100 | 0 |
| 91.04 | neg | 0.02611 | AST | Glycerol | Monosaccharides and metabolites | 1/100 | 0 |
| 111.0225 | pos | 0.02522 | AST |  |  | 1/100 | 0 |
| 115.075 | pos | 0.04912 | HCA | 4-Hydroxy-2-hexenal | Aldehydes | 1/100 | 0 |
| 116.106 | pos | 0.01230 | HCA |  |  | 1/100 | 0 |
| 125.0385 | pos | 0.00825 | AST |  |  | 1/100 | 0 |
| 135.101 | pos | 0.04653 | HCA |  |  | 1/100 | 0 |
| 136.096 | pos | 0.03493 | HCA |  |  | 1/100 | 0 |
| 152.0915 | pos | 0.00883 | HCA |  |  | 1/100 | 0 |
| 173.1345 | pos | 0.00766 | HCA |  |  | 1/100 | 0 |
| 174.0005 | pos | 0.04922 | HCA |  |  | 1/100 | 0 |
| 183.0635 | pos | 0.01948 | AST | 3-Oxo-3-phenylpropanoate | Ethylbenzene degradation, degradation of aromatic compounds | 1/100 | 0 |
| 184.1325 | pos | 0.03356 | HCA |  |  | 1/100 | 0 |
| 194.1385 | pos | 0.02644 | HCA |  |  | 1/100 | 0 |
| 199.049 | pos | 0.00155 | AST | 2,4-Dinitrophenylhydrazine |  | 1/100 | 0 |
| 302.305 | pos | 0.00929 | HCA | Octadecanamide | Fatty amides | 1/100 | 0 |
| 253.2155 | pos | 0.00078 | HCA | 4-Hydroxy-2,6-hexadecadienal | Aldehydes | 0/100 | 1 |
| 262.237 | pos | 0.00076 | HCA |  |  | 0/100 | 1 |
| 272.229 | pos | 0.00064 | HCA |  |  | 0/100 | 1 |
| 274.1285 | pos | 0.00625 | HCA |  |  | 0/100 | 1 |
| 276.145 | pos | 0.00016 | HCA |  |  | 0/100 | 1 |
| 279.231 | pos | 0.00004 | HCA | (13S)-Hydroxyoctadecadienoic acid (13(S)-HODE);  (12R,13S)-(9Z)-12,13-Epoxyoctadecenoic acid (12(13)-EpOME);  (9R,10S)-(12Z)-9,10-Epoxyoctadecenoic acid (9(10)-EpOME) | Linoleic acid metabolism | 0/100 | 1 |
| 281.2475 | pos | 0.00001 | HCA | Linoleate;  Mangiferic acid | Linoleic acid metabolism | 0/100 | 1 |
| 289.2565 | pos | 0.00001 | HCA |  |  | 0/100 | 1 |
| 290.233 | pos | 0.00005 | HCA |  |  | 0/100 | 1 |
| 290.2685 | pos | 0.00000 | HCA |  |  | 0/100 | 1 |
| 291.272 | pos | 0.00018 | HCA |  |  | 0/100 | 1 |
| 295.225 | pos | 0.00021 | HCA | (9Z,11E)-(13S)-13-Hydroperoxyoctadeca-9,11-dienoic acid (13(S)-HPODE) | Linoleic acid metabolism | 0/100 | 1 |
| 300.2585 | pos | 0.00004 | HCA |  |  | 0/100 | 1 |
| 302.2325 | pos | 0.00016 | HCA |  |  | 0/100 | 1 |
| 314.269 | pos | 0.00001 | HCA |  |  | 0/100 | 1 |
| 317.288 | pos | 0.00003 | HCA |  |  | 0/100 | 1 |
| 330.264 | pos | 0.00001 | HCA |  |  | 0/100 | 1 |

*m/z*: mass-to-charge ratio. Charge: ionization mode (either positive or negative). p-values were corrected for multiple hypothesis testing. Elevated in: asthmatic (AST) or healthy control (HCA) with the higher average intensity of the feature. Compounds and chemical families: identified features in this work; for each compound only pathways / chemical families were listed which occurred several times within the study cohort and, thus, were labelled relevant (“..”: no data, several entries: multiple options for compounds associated with the same metabolic pathway). Selection frequency: number of times feature was selected in LOOCV. Final model: features selected by Boruta feature selection method on the full data set for the ﬁnal prediction model.

# References

Benjamini, Y., and Hochberg, Y. (1995). Controlling the False Discovery Rate: A Practical and Powerful Approach to Multiple Testing. *J. R. Stat. Soc. Series B Stat. Methodol. (Methodological)* 57, 289–300.

Buja, A., and Eyuboglu, N. (1992). Remarks on Parallel Analysis. *Multivariate Behav. Res.* 27, 509–540. doi: 10.1207/s15327906mbr2704_2.

Cortes, C., and Vapnik, V. (1995). Support-vector networks. *Mach. Learn.* 20, 273–297. doi: 10.1007/BF00994018.

Dührkop, K., Fleischauer, M., Ludwig, M., Aksenov, A. A., Melnik, A. v, Meusel, M., et al. (2019). SIRIUS 4: a rapid tool for turning tandem mass spectra into metabolite structure information. *Nat. Methods* 16, 299–302. doi: 10.1038/s41592-019-0344-8.

Fawcett, T. (2006). An introduction to ROC analysis. *Pattern. Recognit. Lett.* 27, 861–874. doi: 10.1016/j.patrec.2005.10.010.

Fritsch, F. N., and Carlson, R. E. (1980). Monotone Piecewise Cubic Interpolation. *SIAM J Numer. Anal.* 17, 238–246. doi: 10.1137/0717021.

Jaffe, A. E., Hyde, T., Kleinman, J., Weinbergern, D. R., Chenoweth, J. G., McKay, R. D., et al. (2015). Practical impacts of genomic data “cleaning” on biological discovery using surrogate variable analysis. *BMC Bioinform.* 16, 372. doi: 10.1186/s12859-015-0808-5.

Juniper, E. F., O’Byrne, P. M., Guyatt, G. H., Ferrie, P. J., and King, D. R. (1999). Development and validation of a questionnaire to measure asthma control. *Eur. Respir. J.* 14, 902.

Kaeslin, J., Micic, S., Weber, R., Müller, S., Perkins, N., Berger, C., et al. (2021). Differentiation of Cystic Fibrosis-Related Pathogens by Volatile Organic Compound Analysis with Secondary Electrospray Ionization Mass Spectrometry. *Metabolites* 11, 773. doi: 10.3390/metabo11110773.

Kai, D., Huibin, S., Marvin, M., Juho, R., and Sebastian, B. (2015). Searching molecular structure databases with tandem mass spectra using CSI:FingerID. *Proc. Natl. Acad. Sci.* 112, 12580–12585. doi: 10.1073/pnas.1509788112.

Kanehisa, M., and Goto, S. (2000). KEGG: kyoto encyclopedia of genes and genomes. *Nucleic Acids Res.* 28, 27–30. doi: 10.1093/nar/28.1.27.

Kessner, D., Chambers, M., Burke, R., Agus, D., and Mallick, P. (2008). ProteoWizard: open source software for rapid proteomics tools development. *Bioinformatics* 24, 2534–2536. doi: 10.1093/bioinformatics/btn323.

Kursa, M. B., Jankowski, A., and Rudnicki, W. R. (2010). Boruta – A System for Feature Selection. *Fundam. Inform.* 101, 271–285. doi: 10.3233/FI-2010-288.

Leek, J. T., Johnson, W. E., Parker, H. S., Jaffe, A. E., and Storey, J. D. (2012). The sva package for removing batch effects and other unwanted variation in high-throughput experiments. *Bioinformatics* 28, 882–883. doi: 10.1093/bioinformatics/bts034.

Leek, J. T., and Storey, J. D. (2007). Capturing Heterogeneity in Gene Expression Studies by Surrogate Variable Analysis. *PLoS Genet.* 3, 1724–35. doi: 10.1371/journal.pgen.0030161.

Leek, J. T., and Storey, J. D. (2008). A general framework for multiple testing dependence. *Proc. Natl. Acad. Sci.* 105, 18718–18723. doi: 10.1073/pnas.0808709105.

Li, S., Park, Y., Duraisingham, S., Strobel, F. H., Khan, N., Soltow, Q. A., et al. (2013). Predicting Network Activity from High Throughput Metabolomics. *PLoS Comput. Biol.* 9, e1003123-. doi: 10.1371/journal.pcbi.1003123.

Lin, H.-T., Lin, C.-J., and Weng, R. C. (2007). A note on Platt’s probabilistic outputs for support vector machines. *Mach. Learn.* 68, 267–276. doi: 10.1007/s10994-007-5018-6.

Pang, Z., Chong, J., Zhou, G., de Lima Morais, D. A., Chang, L., Barrette, M., et al. (2021). MetaboAnalyst 5.0: narrowing the gap between raw spectra and functional insights. *Nucleic Acids Res.* 49, W388–W396. doi: 10.1093/nar/gkab382.

Parker, H. S., Corrada Bravo, H., and Leek, J. T. (2014). Removing batch effects for prediction problems with frozen surrogate variable analysis. *PeerJ* 2, e561–e561. doi: 10.7717/peerj.561.

Phipson, B., Lee, S., Majewski, I. J., Alexander, W. S., and Smyth, G. K. (2016). Robust hyperparameter estimation protects against hypervariable genes and improves power to detect differential expression. *Ann. Appl. Stat.* 10, 946–963. doi: 10.1214/16-AOAS920.

Ritchie, M. E., Phipson, B., Wu, D., Hu, Y., Law, C. W., Shi, W., et al. (2015). limma powers differential expression analyses for RNA-sequencing and microarray studies. *Nucleic Acids Res.* 43, e47–e47. doi: 10.1093/nar/gkv007.

Schymanski, E. L., Jeon, J., Gulde, R., Fenner, K., Ruff, M., Singer, H. P., et al. (2014). Identifying Small Molecules via High Resolution Mass Spectrometry: Communicating Confidence. *Environ. Sci. Technol.* 48, 2097–2098. doi: 10.1021/es5002105.

Smyth, G. K. (2004). Linear Models and Empirical Bayes Methods for Assessing Differential Expression in Microarray Experiments. *Stat. Appl. Genet. Mol. Biol.* 3. doi: 10.2202/1544-6115.1027.

Varma, S., and Simon, R. (2006). Bias in error estimation when using cross-validation for model selection. *BMC Bioinform.* 7, 91. doi: 10.1186/1471-2105-7-91.

Weber, R., Haas, N., Baghdasaryan, A., Bruderer, T., Inci, D., Micic, S., et al. (2020). Volatile organic compound breath signatures of children with cystic fibrosis by real-time SESI-HRMS. *ERJ Open Res.* 6, 00171–02019. doi: 10.1183/23120541.00171-2019.
